# Prefrontal engrams of long-term fear memory perpetuate pain perception

**DOI:** 10.1038/s41593-023-01291-x

**Published:** 2023-04-06

**Authors:** Alina Stegemann, Sheng Liu, Oscar Andrés Retana Romero, Manfred Josef Oswald, Yechao Han, Carlo Antonio Beretta, Zheng Gan, Linette Liqi Tan, William Wisden, Johannes Gräff, Rohini Kuner

**Affiliations:** 1 Institute of Pharmacology, Heidelberg University, Heidelberg, Germany; 2 Department of Life Sciences, Faculty of Natural Sciences, Imperial College London, London, UK; 3 Laboratory of Neuroepigenetics, Brain Mind Institute, School of Life Sciences, École Polytechnique Fédérale de Lausanne (EPFL), Lausanne, Switzerland

**Keywords:** Neural circuits, Fear conditioning

## Abstract

A painful episode can lead to a life-long increase in an individual’s experience of pain. Fearful anticipation of imminent pain could play a role in this phenomenon, but the neurobiological underpinnings are unclear because fear can both suppress and enhance pain. Here, we show in mice that long-term associative fear memory stored in neuronal engrams in the prefrontal cortex determines whether a painful episode shapes pain experience later in life. Furthermore, under conditions of inflammatory and neuropathic pain, prefrontal fear engrams expand to encompass neurons representing nociception and tactile sensation, leading to pronounced changes in prefrontal connectivity to fear-relevant brain areas. Conversely, silencing prefrontal fear engrams reverses chronically established hyperalgesia and allodynia. These results reveal that a discrete subset of prefrontal cortex neurons can account for the debilitating comorbidity of fear and chronic pain and show that attenuating the fear memory of pain can alleviate chronic pain itself.

## Abstract (web summary)

Can erasing bad memories relieve pain? Stegemann et al. uncover the cellular basis of fear–pain interactions, reporting that fear potentiates pain via memories encoded in prefrontal engrams. Blocking these memory traces reduces chronic pain in mice.

## Main

Pain and fear are independent behavioral states that are interrelated in a dichotomous manner^1,2^. In the face of danger, fear acutely suppresses pain perception^2^, which is critical for survival; this phenomenon can be experimentally mimicked: acute fear induction in response to a highly painful stimulus results in short-lasting analgesia to subsequent noxious stimuli. This ‘fear-conditioned analgesia’ is well studied, involves opioidergic and endocannabinoidergic mechanisms and is mediated by recruitment of bulbospinal descending pathways that inhibit spinal transfer of nociceptive information^3^. However, long-term associative fear memory induced by previous exposure to pain has also been proposed to serve as a critical predisposing factor for pain chronicity, and fear of pain can elicit avoidance behaviors and exacerbate pain^1,3–5^. The neurobiological basis of this interaction between long-term associative fear memory and pain, particularly in a chronic context, is of paramount biological and clinical significance but is poorly understood. The present study addresses this gap by tagging and manipulating engrams, that is, physical traces of memory^6^, which have been suggested to form the functional substrate for long-term storage of cognitive associations at the cellular level^7^.

## Results

### Activity-dependent tagging of prefrontal engrams in long-term fear and pain

**Fig. 1:**
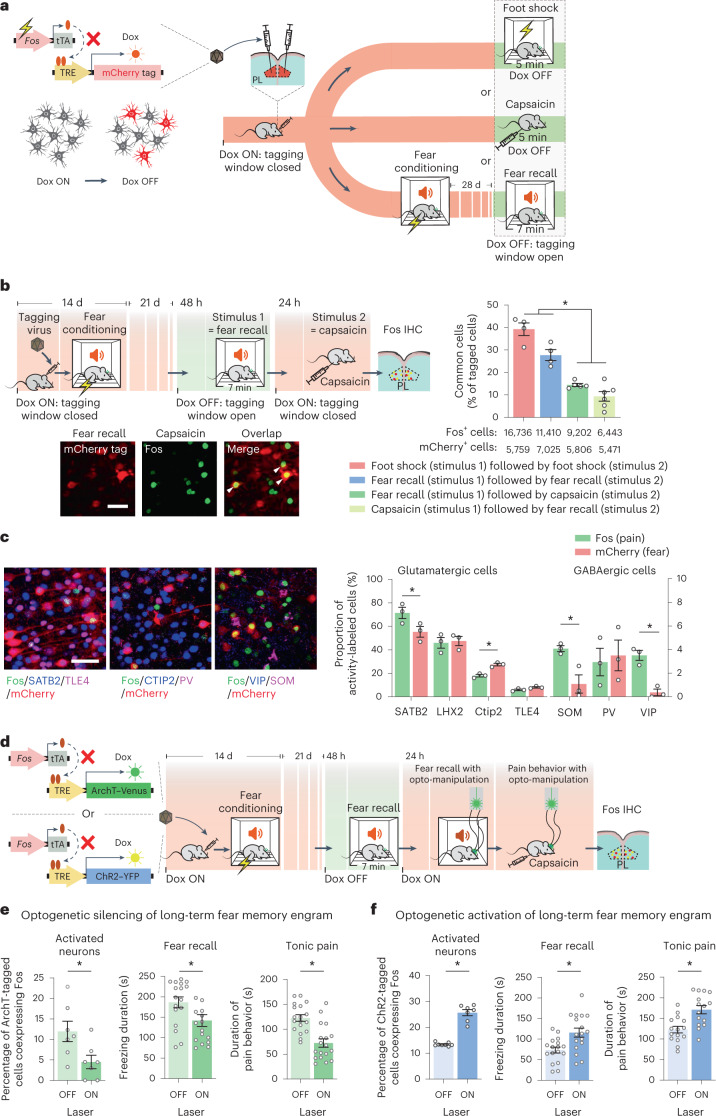
Anatomical prefrontal substrates and functional interrogation of the interplay between long-term memory and tonic pain. **a**, Viral-mediated, Dox-controlled expression of protein tags under the *Fos* promoter, leading to activity-dependent tagging of prelimbic neurons with mCherry following painful foot shock or capsaicin or during recall of fear memory 28 d after cued fear conditioning. **b**, Experimental scheme (top left), typical examples (bottom left) showing successive labeling of neuronal populations over discrete behavioral states with mCherry and Fos immunohistochemistry (arrowheads: double-labeled neurons; scale bar, 50 µm) and quantitative summary (top right) of overlapping activated neuronal populations; total numbers of positive/tagged cells are shown underneath. Data were analyzed by one-way ANOVA with Tukey’s multiple comparisons test; *P < 0.05; foot shock followed by foot shock and fear recall followed by fear recall, *n* = 4 mice per group; fear recall followed by capsaicin, *n* = 5 mice per group; capsaicin followed by fear recall, *n* = 6 mice per group; IHC, immunohistochemistry. **c**, Characterization of labeled prefrontal neurons in fear recall engrams or by tonic pain using markers of excitatory and GABAergic neurons. Typical examples (left) and a quantitative summary (right) are shown; scale bar, 50 µm. Data were analyzed by two-way ANOVA with a Šidák correction for multiple comparisons test; *P < 0.05; *n* = 3 mice per group; PV, parvalbumin. **d**–**f**, Experimental scheme (**d**) for activity-dependent tagging of long-term fear engrams with optogenetic actuators ArchT or ChR2 and testing effects of light-induced silencing (**e**) or activation (**f**) of fear engrams on prelimbic Fos immunohistochemistry, fear recall behavior and capsaicin-evoked tonic pain-related behavior are shown. In **e** and **f**, an average of 4,280 neurons per mm^3^ were labeled with ArchT and 3,894 neurons per mm^3^ were labeled with ChR2, respectively; light-induced silencing and activation: unpaired *t*-test, *n* = 7 mice per group (**e**) and laser off *n* = 7 mice per group and laser on *n* = 8 mice per group (**f**); fear recall behavior: paired *t*-test, *n* = 17 mice (**e**) and *n* = 17 mice (**f**); tonic pain-related behavior: paired *t*-test, *n* = 18 mice (**e**) and *n* = 15 mice (**f**). All *P* and *F* values are shown in Supplementary Table 1. Source data

**Extended Data Fig. 1:**
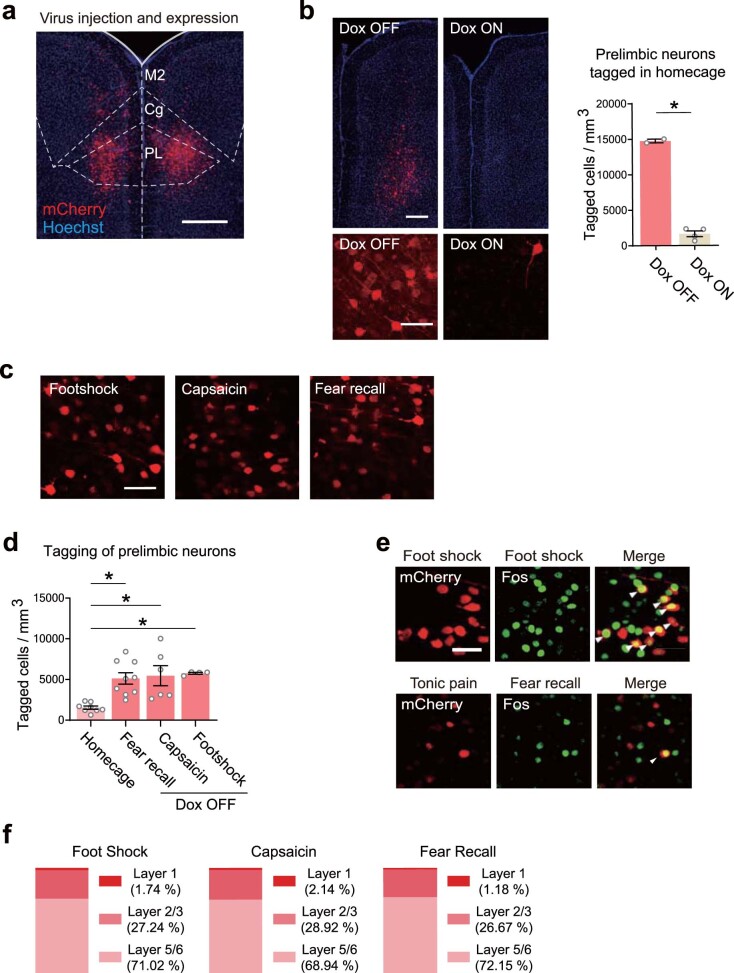
Activity labeling system for labelling prefrontal engrams for long term fear recall and neurons responsive to foot shock stimulation and tonic pain. **(a)** Doxycycline-controlled, activity-evoked expression of protein tags under *c-fos* promoter following viral injection of tagging constructs in the murine prelimbic cortex. Shown is an example of neurons tagged with mCherry expression in an activity-dependent manner. (**b**) Control of tagging by presence of Doxycyline (Dox ON, 1 mg/mL Dox for 16 days) in comparison to Doxycline-free time window (Dox OFF) is demonstrated in baseline home-cage conditions. Example images show mCherry reporter expression (red) on the background of Hoechst-stained cell nuclei (blue). Magnified images are shown in lower panels. Quantitative summary is shown on the right; unpaired t-test; * *p* < 0.05; Dox OFF: n = 2 mice/group, Dox ON: n = 4 mice/group. (**c, d**) Examples (panel c) and quantitative summary (panel d) of prelimbic neuronal labeling upon foot shock, fear memory recall at 28 days post-conditioning or paw injection of capsaicin. In panel d, one-way ANOVA with Dunnet’s multiple comparisons; * *p* < 0.05; Homecage: n = 7 mice/group, Fear Recall: n = 9 mice/group, Capsaicin: n = 6 mice/group, Footshock: n = 4 mice/group. **(e)** Typical examples showing dual labeling of neuronal activity over two discrete events of the same behavioral state or two distinct behavioral states via mCherry and Fos immunohistochemistry. Arrow-heads: double-labelled neurons. Scale bars represent 0.5 mm in panel a, 250 µm in upper images in panel b and 50 µm lower images in panel b, 100 µm in panel c and 50 µm in panel e. **(f)** Distribution of labelled neurons across layers of the prelimbic cortex. All data are shown as mean + /− S.E.M. All *p* and *F* values are shown in Supplementary Table 1. Source data

Given that the medial prefrontal cortex is one of the most frequently activated brain areas in pain in human imaging studies and animal models^8–10^ and is also closely associated with long-term memory storage and retrieval of fear^7,11,12^, we studied the mouse homolog of the human medial prefrontal cortex, namely, the prelimbic cortex. We used an activity-dependent neural tagging approach^6,13,14^ that relies on doxycycline (Dox) to open (Dox OFF) or close (Dox ON) temporal windows (Fig. 1a and Extended Data Fig. 1a,b; all *P* and *F* values are given in Supplementary Table 1 for all groups). Herein, expression of tetracycline-controlled transactivator (tTA) in the mouse prelimbic cortex under control of the activity-dependent promoter of the immediate early gene *c-fos* enables expression of tags, such as mCherry, via binding of tTA to the tTA-responsive promoter element (TRE; Fig. 1a and Extended Data Fig. 1a), which is inhibited by Dox. Thus, keeping the mice on Dox (Dox ON) represses background expression (Extended Data Fig. 1b), and taking the mice off Dox (Dox OFF) opens a finite time window to label neuronal populations with mCherry expression in response to a given external stimulus or behavioral state (Extended Data Fig. 1c,d).

We used this approach for conditionally ‘capturing’ the prefrontal fear engram during recall of associative fear that was encoded 4 weeks earlier in a fear conditioning paradigm involving painful foot shocks; thus, prefrontal neurons that were specifically activated during long-term recall of fear (as opposed to acquisition of fear) several weeks after fear conditioning were specifically tagged (Extended Data Fig. 1c,d). In other groups of mice that did not undergo fear conditioning, we tagged prefrontal neuronal assemblies activated during aversive and painful behavioral states using foot shock stimulations or hind paw injection of the algogen capsaicin (Extended Data Fig. 1c,d). Neurons participating in the long-term fear memory engram did not differ in their distribution across the prelimbic cortex from neurons activated during capsaicin-induced tonic pain, which builds up acutely and is maintained over several minutes, with the largest number of labeled cells found in prelimbic layers 5 and 6, followed by layers 2 and 3 (Extended Data Fig. 1f). Next, we identified prefrontal neurons that are coactivated during tonic pain and long-term fear memory using a dual labeling approach via use of the aforementioned tagging approach for the first stimulus (for example, fear recall), complemented by Fos immunohistochemistry shortly following a second stimulus (for example, capsaicin; Fig. 1b). We validated this strategy by giving two distinct electroshock stimuli 24 h apart to the hind paw, which led to reactivation of 40% of prefrontal neurons (Fig. 1b and Extended Data Fig. 1e), thus reflecting the relative stability of engrams expected from previous studies^7,11^. We noted that 15% of prefrontal neurons are commonly tagged during fear memory and tonic pain, which comprise about 30% of the cells that are recruited during either state (Fig. 1b), suggesting partly overlapping encoding of pain perception and long-term fear in the prefrontal cortex. This overlap in long-term fear memory engram and pain far exceeded the 1.7% overlap that would be expected by chance when calculated as described previously^7^. In these analyses, overlap was calculated as neurons double positive for Fos and mCherry as a percent proportion of total mCherry-labeled neurons. Similar observations were made after representing the data in the form of double-positive neurons as a function of total Fos-expressing cells.

Prelimbic neurons labeled by increased activity during fear recall or pain were detected in all prefrontal layers 5 and 6, which are important in prefrontal output and harbored the largest proportion of labeled neurons, followed by layers 2 and 3, which are important in processing and cortico-cortical associative connectivity^15,16^ (Extended Data Fig. 1f). Coimmunohistochemical analysis with characteristic markers of excitatory and inhibitory neurons^15,16^ revealed that pain-responsive neurons entail higher proportions of cortico-cortically projecting (SATB2-expressing) excitatory neurons and two classes of inhibitory GABAergic neurons (somatostatin (SOM) and vasoactive intestinal peptide (VIP)) than fear memory engrams (Fig. 1c), thus showing the distinct composition of these neuronal cohorts. We also tested the identity of neurons that are commonly activated in the fear memory engram and pain and observed that they comprise an overwhelmingly large population of excitatory cells, as inhibitory neurons comprise less than 2% (parvalbumin neurons: 1.6%; VIP neurons: 0.3%; SOM neurons: 0%). The largest proportion of the overlap population was given by cortico-cortically projecting SATB2-expressing neurons (90%), followed by LHX2^+^ (66%), CTIP2^+^ (37%) and TLE4^+^ (6%).

### Impact of optogenetic manipulation of prefrontal fear engrams on pain and specificity controls

**Extended Data Fig. 2:**
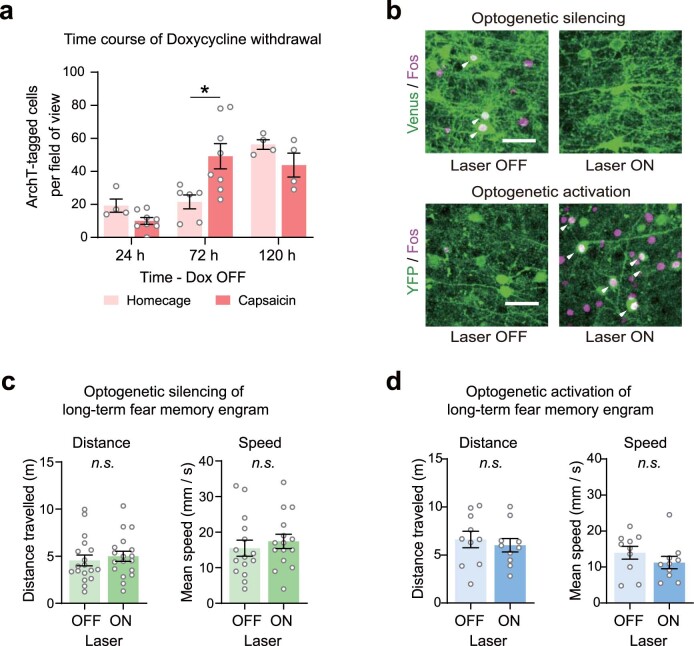
Control experiments for optogenetic activation or silencing of prefrontal fear memory engram. **(a)** Optimization of the time course of Doxycycline (Dox) withdrawal to uncover conditions yielding a significant increase (* *p* < 0.05) in the number of cells labelled with optogenetic actuators, when compared to homecage controls (two-way ANOVA with Sidak’s test for multiple comparisons); Capsaicin: n = 4 injections/group, 72 h Homecage: n = 6 injections/group, 24 h Capsaicin/72 h Capsaicin: n = 8 injections/group. **(b)** Typical examples of labeling prefrontal remote fear memory engram neurons with Channelrhodopsin (ChR2-YFP) or Archeorhodopsin (ArchT-Venus) and validation of optogenetic activation or silencing, respectively, via increase or decrease in immunohistochemical detection of expression of activity marker Fos respectively, upon laser light stimulation *in vivo*; arrowheads indicate double-labelled neurons and scale bars represent 50 µm. (**c, d**) Behavior associated with locomotion in open field (in the same cohort of mice with (Laser ON) or without (Laser OFF)) upon optogenetic silencing (c) or activation (d) of the prefrontal fear memory engram; paired *t*-test; In panel c, distance travelled: n = 18 mice/group, speed: n = 15 mice/group; in panel d, n = 10 mice/group; n.s.: non-significant. All data are shown as mean + /− S.E.M. All *p* and *F* values are shown in Supplementary Table 1. Source data

To assess the functional meaning of this overlap, we next tagged the prefrontal engram of long-term fear memory with the optogenetic inhibitory opsin archeorhodopsin (ArchT; Fig. 1d,e and Extended Data Fig. 2a,b). The Dox ON/Dox OFF dynamics and dosage were technically optimized to yield specific ArchT expression (Extended Data Fig. 2a) and ArchT-mediated silencing in vivo, as verified via Fos expression (Fig. 1e and Extended Data Fig. 2b), paving the way for functional behavioral analyses. When ArchT was optogenetically activated after remote fear memory recall, we found that the activity of the fear memory engram and fear memory recall behavior were reduced, as expected from previous studies^7^. Surprisingly, in a different session that assessed the impact of ArchT-mediated silencing of the fear recall-labeled neurons on pain, we also observed robustly suppressed voluntary pain-related behaviors, such as flicking, lifting and licking of the affected paw (Fig. 1e and Supplementary Videos 1 and 2). Conversely, activating the prefrontal neurons tagged during fear recall with the excitatory optogenetic actuator channelrhodopsin–yellow fluorescent protein (ChR2–YFP; Fig. 1f and Extended Data Fig. 2b) was sufficient to evoke fear-related freezing behavior even in the absence of auditory and contextual conditioned cues, thus demonstrating their validity as ‘long-term fear memory engram’ neurons. Paradoxically, activating this engram also markedly exacerbated capsaicin-induced tonic pain behaviors (Fig. 1f). These results thus unexpectedly reveal that the percept of tonic, ongoing pain contains profound traces of a long-term fear memory from a prior pain experience.

**Fig. 2:**
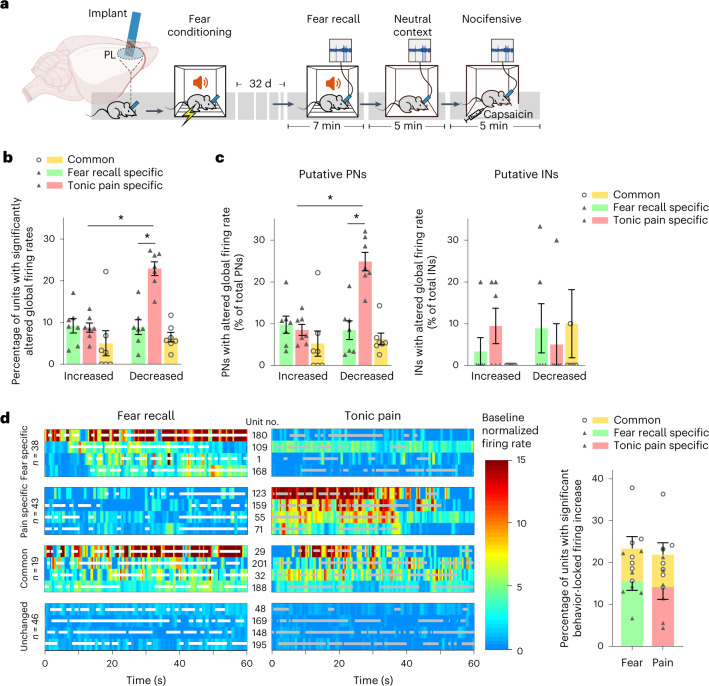
In vivo tetrode recordings to study encoding of fear recall and tonic pain in prefrontal excitatory neurons and inhibitory neurons. **a**, Schematic representation of experiments on electrophysiological recordings using tetrodes implanted in the prelimbic cortex (PL) during fear recall, neutral context or capsaicin-induced tonic pain. **b**, Summary of prelimbic units (*n* = 261 units from seven animals) demonstrating significant rate changes during fear recall or tonic pain over the neutral context. **c**, Delineation of global rate-coding units for fear recall and tonic pain into putative principal (excitatory) neurons (PN; left; *n* = 231 units) and interneurons (IN; right; *n* = 30 units). Data were analyzed by two-way ANOVA with a Šidák correction for multiple comparisons test; *P < 0.05; *n* = 7 mice per group. Data are shown as mean ± s.e.m. **d**, Example heat map illustrations (left) and quantitative summary (right) of data from in vivo prelimbic tetrode recordings showing rate-coding units with temporal specificity for episodes (superimposed on units) of fear-related freezing behavior or pain-related nocifensive behaviors, activity common to both or no association with either behavior. The proportion of behavior-specific firing-increased neurons, detected with a permutation test for mean difference between binned firing rates (*P* < 0.05, effect size > 0.5; *n* = 146 units over seven mice), was similar in fear recall and tonic pain states (*P* > 0.5, unpaired *t*-test). Shaded horizontal lines represent state-specific behavioral episodes. All data are shown as mean ± s.e.m. All *P* and *F* values are shown in Supplementary Table 1. Source data

**Extended Data Fig. 3:**
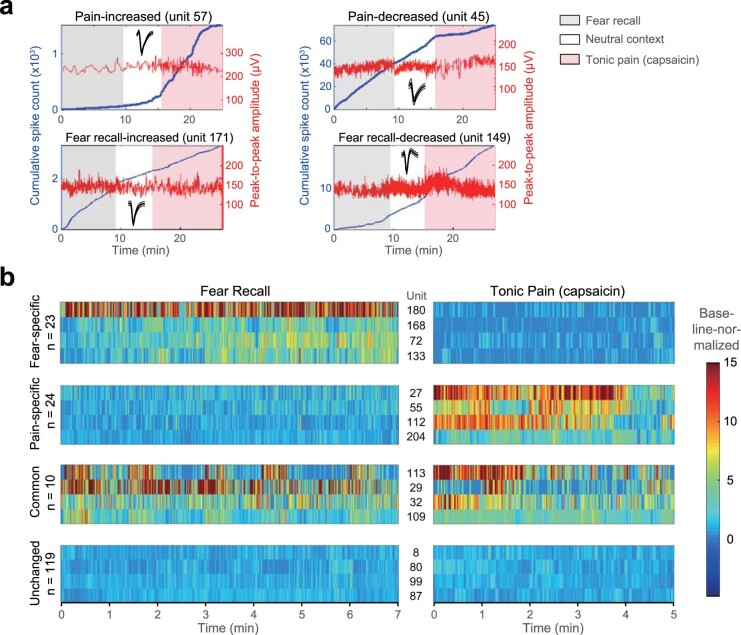
*In vivo* tetrode recordings to study encoding of fear recall and tonic pain in prefrontal excitatory neurons and inhibitory neurons. **(a)** Example plots showing state-specific changes in firing rates of units. Insets show spike waveforms (mean ± SD, 1.2 ms) for each unit. **(b)** Example heat map illustration of binned firing rates normalized to activity in neutral context for units showing increased activity during fear recall or tonic pain. Source data

We performed additional experiments to validate these inferences and to confirm specificity. First, to control for bias that may be introduced by potential tropism of the adeno-associated viral (AAV) vectors used for cell tagging and the use of Fos expression as a non-linear surrogate parameter for neuronal activity, we performed electrophysiological recordings of prefrontal neuronal firing with high temporal resolution using in vivo tetrodes in awake, behaving mice (Fig. 2a). Activity of 261 prelimbic units was tested in multiple paired recording sessions over phases of remote fear recall (using the same auditory-cued fear conditioning paradigm used in the cell labeling approach described above) and capsaicin-induced tonic pain in a total of seven mice. Analysis of globally altered rates of firing under either fear recall or tonic pain conditions compared to the neutral context confirmed the existence of distinct prefrontal neurons linked to either long-term fear recall or tonic pain (that is, state specific, comprising about two-thirds) and a commonly activated subset (about one-third; Fig. 2a,d and Extended Data Fig. 3a,b); neurons demonstrating an increased firing rate in both pain and fear recall were found in waveform patterns and firing rate analyses to exclusively comprise principal (excitatory) neurons (Fig. 2c). Both excitatory and inhibitory prelimbic neurons showing decreased firing rates in association with fear memory or pain behavior were also observed (Fig. 2b,c). We used simultaneous video monitoring to identify neurons whose activities were temporally phase locked to intermittent behavioral episodes of fear recall (freezing) or pain-induced suffering and escape behaviors (lifting, flicking or licking of the capsaicin-injected paw). This revealed, in addition to state-specific neurons, a significant subset (about 30% of total responsive neurons) that was commonly activated during manifestation of fear memory and perception of ongoing pain (Fig. 2d), similar to our findings from viral, *c-fos* promoter-based activity-mapping experiments.

**Fig. 3:**
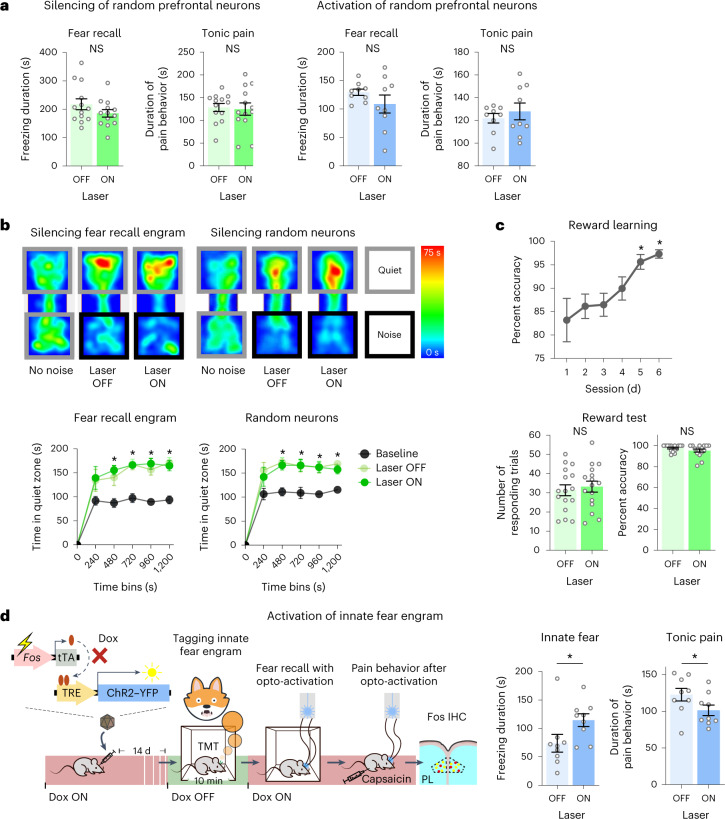
Analysis of specificity of the interplay between long-term fear memory and pain. **a**, Impact of optogenetic silencing or activation of a randomly targeted prelimbic neuronal population on long-term fear recall and capsaicin-induced nocifensive behaviors; an average of 6,919 neurons per mm^3^ were labeled with ArchT and 3,689 neurons per mm^3^ were labeled with ChR2. Data were analyzed by paired *t*-test; *n* = 13 mice for the silencing group and 9 mice for the activation group. **b**, Impact of optogenetic silencing of the fear engram or random prefrontal neurons on avoidance of non-painful, aversive white noise, shown in the form of example body heat maps (above) and quantitative overview (below). Data were analyzed by two-way ANOVA with a Šidák correction for multiple comparisons; *P < 0.05 compared to baseline; silencing fear recall neurons: *n* = 10 mice per group; silencing random neurons: *n* = 11 mice per group. **c**, Impact of optogenetic silencing of prefrontal fear engram neurons on appetitive reward learning behavior. For reward learning, a one-way ANOVA with a Dunnett correction for multiple comparisons was performed. For the number of responding trials, a paired *t*-test was performed and for percent accuracy a Wilcoxon test was performed; *P < 0.05; *n* = 16 mice per group. **d**, Experimental scheme for labeling prefrontal neurons activated during recall of innate fear and impact of their optogenetic activation on fear recall and capsaicin-induced tonic pain behavior. Data were analyzed by paired *t*-test; *P < 0.05; *n* = 9 mice per group; NS, not significant. All data are shown as mean ± s.e.m. All *P* and *F* values are shown in Supplementary Table 1. Source data

**Extended Data Fig. 4:**
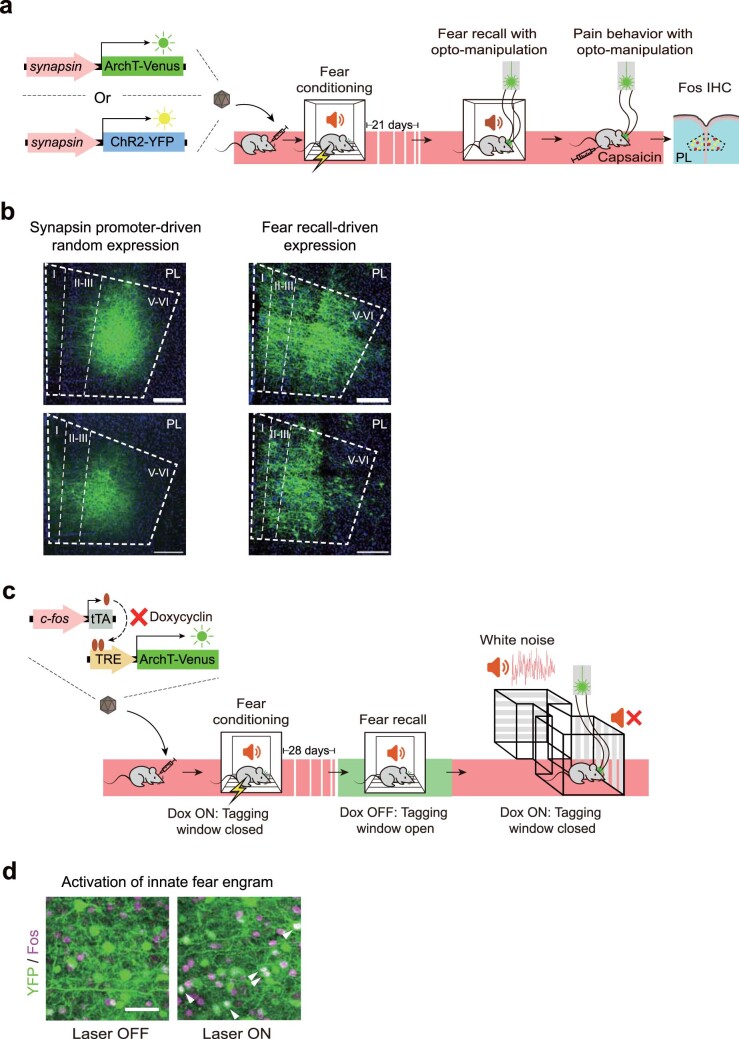
Experiments for testing specificity of interaction between fear memory and pain. (**a**) Experimental scheme for labelling of a random population of prelimbic neurons with optogenetic actuators ArchT and ChR2 using the murine *synapsin* promoter to drive expression, and scheme for testing effect of their activation or silencing on remote fear recall and capsaicin-induced pain behaviors. **(b)** Examples of reporter expression driven by random labelling of neurons using the synapsin promoter or expression driven by labelling of the fear recall engram in the prelimbic cortex; scale bars represent 200 µm. The injection was repeated and verified in n = 6 mice in the Synapsin promotor-driven expression group and n = 8 in the fear recall-driven expression group. (**c**) Experimental scheme for testing impact of optogenetic silencing of the prefrontal fear memory engram on pain-unrelated aversion to white noise. (**d**) Typical examples of labeling prefrontal neurons responding to innate fear upon exposure to compound TMT from fox urine with Channelrhodopsin (ChR2-YFP) and validation of optogenetic activation via increase in immunohistochemical detection of Fos upon laser light stimulation *in vivo*; arrowheads indicate double-labelled neurons and scale bars represent 50 µm. Source data

Second, suppression of ongoing pain by optogenetic inhibition of the prefrontal fear recall engram did not arise from confounding motor dysfunction, as movements and locomotion were unimpaired (Extended Data Fig. 2c,d). Third, to test whether the phenotypic changes were the result of non-specific blockade of prefrontal activity, we expressed ArchT randomly using the mouse synapsin promoter in prelimbic neurons (Extended Data Fig. 4a), establishing conditions comparable to ArchT expression in fear engrams (Extended Data Fig. 4b). Optogenetic inhibition of a random population of prefrontal neurons suppressed neither fear recall nor pain (Fig. 3a and Extended Data Fig. 4a,b).

We next tested the specificity of the observed interaction between chronic fear and pain by assessing whether negative or positive valence unrelated to pain were affected. For the former, we used aversive white noise in a modified real-time place avoidance paradigm, in which mice increasingly avoided a chamber coupled with aversive white noise, spending more time in the chamber with less noise (Fig. 3b and Extended Data Fig. 4c). Optogenetic suppression of the prefrontal long-term fear engram did not elicit a change in avoidance behaviors that reflected pain-unrelated aversion (Fig. 3b). To test pain-unrelated positive valence, we used an operant conditioning paradigm in which water-restricted mice learned to seek a water reward in a touch screen chamber. We ascertained that despite a period of 8 d between the last day of the learning curve and operant testing, control mice maintained a high level of accuracy. Mice with ArchT expression in the prefrontal fear recall engram showed equivalent operant responsivity and task accuracy in the presence or absence of yellow illumination (Fig. 3c). Thus, optogenetic inhibition of the remote fear memory engram did not generally affect conditioned learning and prefrontal contributions in aversive or appetitive functions, thereby underlining the specificity of the interplay between associative fear memory and pain perception via prefrontal mechanisms.

Is the exacerbation of tonic pain specific for long-term fear memories that are encoded by fear of pain? To address this question, we tested the impact of optogenetic tagging and activation of a prefrontal engram on innate fear^17^ of a predator using exposure to 2,3,5-trimethyl-3-thiazoline (TMT), a compound found in fox urine, as a surrogate for the predator (Fig. 3d and Extended Data Fig. 4d). Optogenetic activation of the tagged population at a later time point induced freezing behavior, reflecting recall of innate fear (Fig. 3d). Rather than being exacerbated, tonic pain was suppressed to a small but significant extent (Fig. 3d), suggesting that the interaction is similar to that seen with acute stress, which induces analgesia^3^. This further underlines the specificity of pain aggravation by long-term associative memory induced by a previous painful episode.

### Alterations of fear–pain interactions under chronic pain conditions

**Fig. 4:**
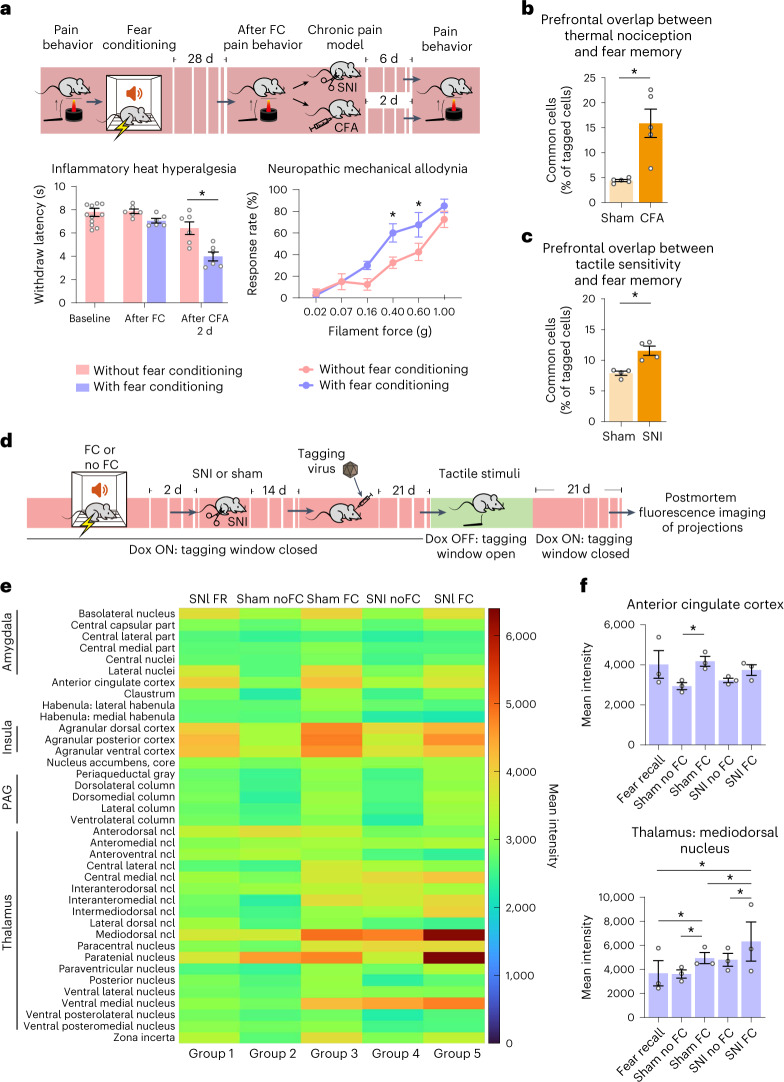
Plasticity of prefrontal representation of fear memory, nociception and touch and their connectivity in inflammatory and neuropathic pain. **a**, Experimental scheme (top) and impact of fear conditioning on withdrawal responses of the contralateral paw to heat or to graded intensities of mechanical stimulation in naive mice (baseline) and mice with unilateral paw inflammation (CFA; *n* = 6 mice per group) or nerve injury (SNI; *n* = 8 mice per group). Data were analyzed by two-way ANOVA with a Tukey’s multiple comparisons test; *P < 0.05; FC, fear conditioning. **b**, Quantitative summary of overlapping prefrontal neurons commonly activated by a heat ramp (Fos) and long-term fear memory (ArchT–Venus) under baseline conditions (average Fos^+^ cells = 5,935 cells per mm^3^; average Venus^+^ cells = 3,028 cells per mm^3^) and after paw inflammation (average Fos^+^ cells = 7,306 cells per mm^3^; average Venus^+^ cells = 2,612 cells per mm^3^). Data were analyzed by unpaired *t*-test; *P < 0.05; *n* = 5 mice per group. **c**, Quantitative summary of overlapping prefrontal neuronal populations commonly activated by tactile stimulation (Fos) and long-term fear memory (ArchT–Venus) under sham (average Fos^+^ cells = 5,400 cells per mm^3^; average Venus^+^ cells = 4,656 cells per mm^3^) and neuropathic conditions (average Fos^+^ cells = 5,011 cells per mm^3^; average Venus^+^ cells = 5,319 per mm^3^). Data were analyzed by unpaired *t*-test; *P < 0.05; *n* = 4 mice per group. **d**–**f**, Scheme for labeling projections (**d**) and analysis of projection intensity (**e**) from prefrontal neurons tagged during tactile stimulation under baseline conditions (sham) and following SNI in mice subjected to fear conditioning or not. Projection density is shown in the form of heat maps of tactile-responsive prefrontal neurons (groups 2–5) that were either not exposed to fear conditioning (groups 2 and 4) or following fear conditioning (groups 3 and 5) under sham conditions (groups 2 and 3) and after SNI (groups 4 and 5); SNI FR, projection maps from prefrontal fear engram neurons labeled during long-term fear recall (group 1); *n* = 3 mice per group. PAG, periaqueductal gray. Data in **f** show average values of projection intensity in the rostral anterior cingulate cortex and mediodorsal thalamic nucleus. Data were analyzed by two-way ANOVA with a Šidák test for multiple comparisons; *P < 0.05. All data are shown as mean ± s.e.m. All *P* and *F* values are shown in Supplementary Table 1. Source data

**Extended Data Fig. 5:**
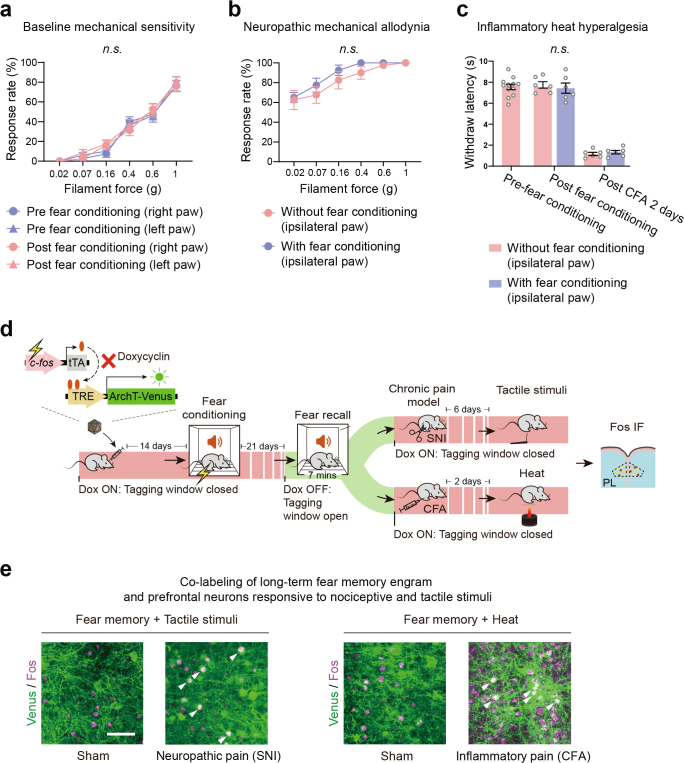
Impact of cued fear conditioning on nociceptive and tactile sensitivity and prefrontal basis of interplay between fear and pain. **(a)** Impact of fear conditioning on paw withdrawal responses to graded mechanical von Frey force across noxious and non-noxious intensities in naïve mice (baseline); n = 16 mice/group. (**b**) Impact of fear conditioning on withdrawal responses to von Frey hairs after nerve injury; shown are data from the ipsilateral paw; n = 8 mice/group. (**c**) Impact of fear conditioning on withdrawal responses to thermal nociceptive stimuli (Hargreaves plantar test) in baseline condition and longitudinally after induction of unilateral paw inflammation (CFA); shown are data from the ipsilateral paw; n = 6 mice/group. In all panels, two-way ANOVA with Tukey’s multiple comparisons test was performed. **(d)** Experimental scheme for labelling prefrontal long-term fear memory engram and testing its overlap with prefrontal neurons responsive to a heat ramp or tactile von Frey stimulation in baseline conditions and changes thereof after induction of paw inflammation (CFA) or nerve injury (SNI). **(e)** Typical examples fear engram-labelled neurons (Venus) and overlap (arrows) with neurons activated by light touch or heat (Fos+Venus); the expression was verified and quantified in n = 4 mice in the Sham group and n = 4 mice in the SNI group; scale bars represent 50 µm; n.s.: non-significant. Data are shown as mean + /− S.E.M. All *p* and *F* values are shown in Supplementary Table 1. Source data

Thus far, our data show that modulation by long-term fear comes into play during tonic pain and suffering, which necessarily involves cortical circuitry^16^. By contrast, in our hands, subcortically/spinally determined rapid withdrawal behaviors elicited by noxious stimuli, reflecting nociception^16^, were not affected by either fear conditioning (Fig. 4a) or manipulations of prefrontal fear memory engrams in mice under baseline (physiological) conditions (Extended Data Fig. 5a). However, this aspect changed dramatically under conditions of pathological pain. We found evidence that long-term fear memory is linked to the pathological shift in nociceptive sensitivity following tissue injury, which is a key hallmark of pathological pain disorders and is known to be subject to cortical modulation^15^. In mice with unilateral paw inflammation induced by injection of complete Freund’s adjuvant (CFA) and in mice with unilateral peripheral spared nerve injury (SNI), subsequent cued fear conditioning enhanced the rate of withdrawal responses to mechanical force and reduced the latency to withdrawal from heat, thus inducing hypersensitivity at the uninjured contralateral paw (Fig. 4a and Extended Data Fig. 5b,c); changes at the injured paw were not significant, likely due to a ceiling effect. These findings are indicative of a ‘second hit’ scenario, in which tissue injury adds on to a heightened fear of imminent pain from a previous pain exposure, leading to abnormally disproportionate nociceptive hypersensitivity.

**Extended Data Fig. 6:**
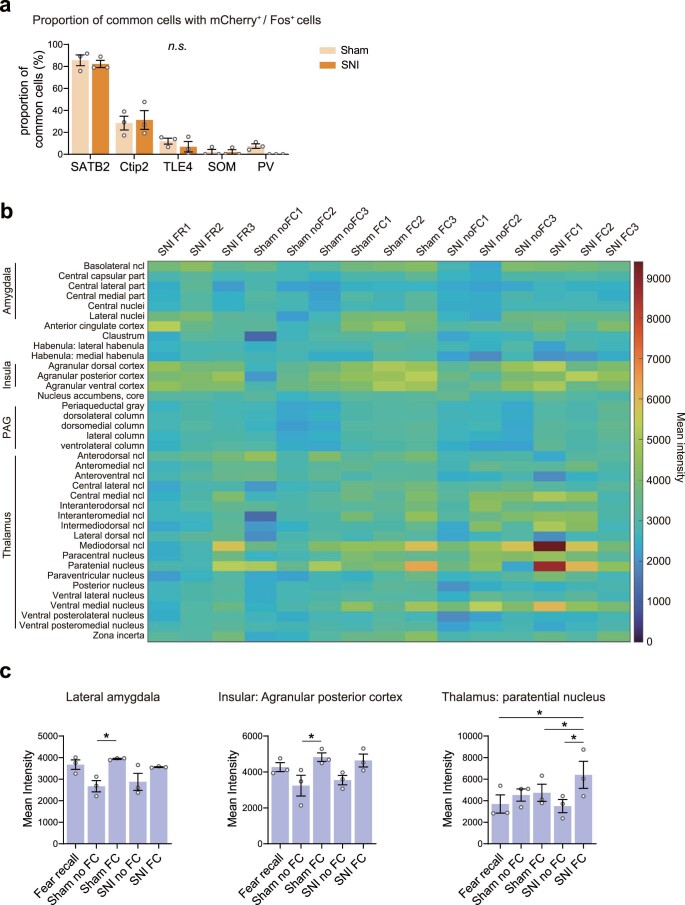
Analysis of neural circuit mechanisms of interactions between long-term fear memory and chronic pain. **(a)** Analysis of neurochemical identity of prelimbic neurons that are activated commonly between long-term fear recall engram (mCherry-expressing) and mechanical pain (Fos-expressing) in mice 4 days after nerve injury (SNI) or sham surgery (sham). Shown is their classification based upon immunohistochemical detection of marker proteins; n = 3 mice/group; two-way ANOVA with Tukey’s multiple comparisons test was performed. Data are shown as mean + /− S.E.M. **(b**) More detailed representation of projection density shown in main Fig. 3e; here, data are shown for individual mice in form of heat maps for tactile-responsive prefrontal neurons (groups 2–5) that were either not exposed to fear conditioning (groups 2 and 4) or following fear conditioning (groups 3 and 5) in physiological conditions (sham, groups 2 and 3) and after nerve injury (SNI, groups 4 and 5); projection heat maps from prefrontal fear engram neurons labelled in mice with long-term fear recall (group 1) are included here comparison; n = 3 mice/group. **(c)** Average values of projection intensity in the indicated areas; this figure extends the data shown in main Fig. 3f. n = 3 mice/group. Two-way ANOVA with Sidak’s test for multiple comparisons, * *p* < 0.05; n.s.: non-significant. All data are shown as mean + /− S.E.M. All *p* and *F* values are shown in Supplementary Table 1. Source data

To test whether this emergent interaction between nociception and fear memory involves prefrontal networks, we performed two independent experiments. First, we studied prefrontal neurons activated during thermal or mechanical stimulation (Extended Data Fig. 5d). Interestingly, the proportion of prefrontal neurons common to engrams of long-term fear memory and heat stimulation was significantly enhanced in mice after induction of inflammatory pain with CFA compared to in mice under baseline conditions (Fig. 4b and Extended Data Fig. 5e). This did not arise from pure chance, as chance overlap between neurons labeled by thermal nociception and fear memory was calculated to be 0.07%. Similarly, prefrontal engrams of long-term fear memory showed very little overlap with prefrontal mechanical (tactile) representation under normal conditions but expanded to demonstrate significantly increased overlap after SNI (Fig. 4c and Extended Data Fig. 5e; chance overlap between neurons labeled by tactile sensitivity and fear memory was 0.03%). However, the proportion of subtypes of excitatory and inhibitory neurons found within the neuronal populations between mechanical representation or fear recall did not change between SNI and sham conditions (Source Data for Extended Data Fig. 6a), nor did the common (overlapping) population between fear recall and tactile sensitivity change after nerve injury (Extended Data Fig. 6a). Because prefrontal neurons responsive to somatosensory inputs and those that participate in the fear memory engrams also did not exhibit obvious differences in localization across the prefrontal cortex in our cell-tagging studies, we next assessed whether they show divergent connectivity properties that could account for differences in their function.

**Extended Data Fig. 7:**
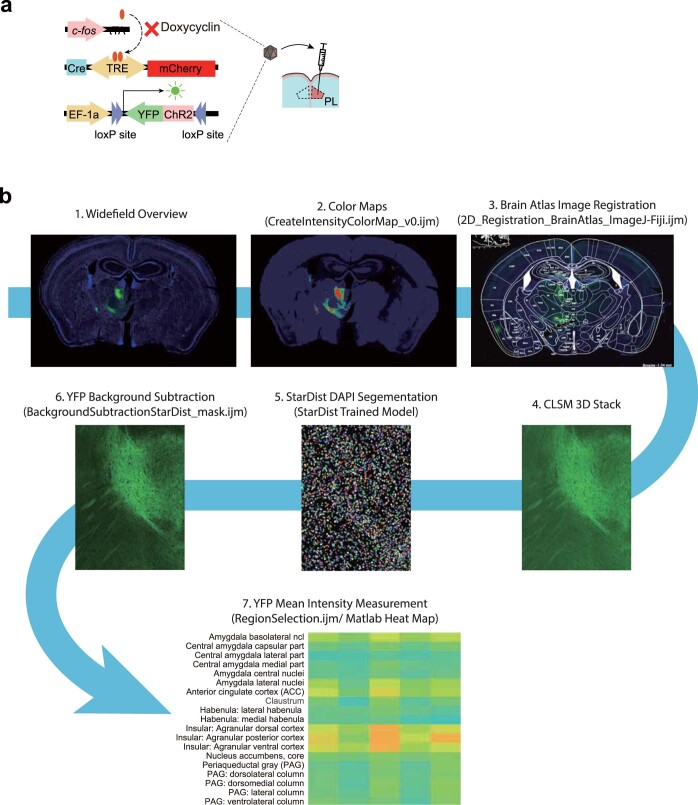
Experimental scheme and analysis strategy for connectivity analysis of specific functional groups of prefrontal neurons. **(a)** Details of viral constructs employed for activity-dependent labeling of prefrontal neurons and their projections. **(b)** Steps undertaken for measuring intensity of projections of labelled neurons to specific brain areas and conversion to heat maps for representation. Source data

**Extended Data Fig. 8:**
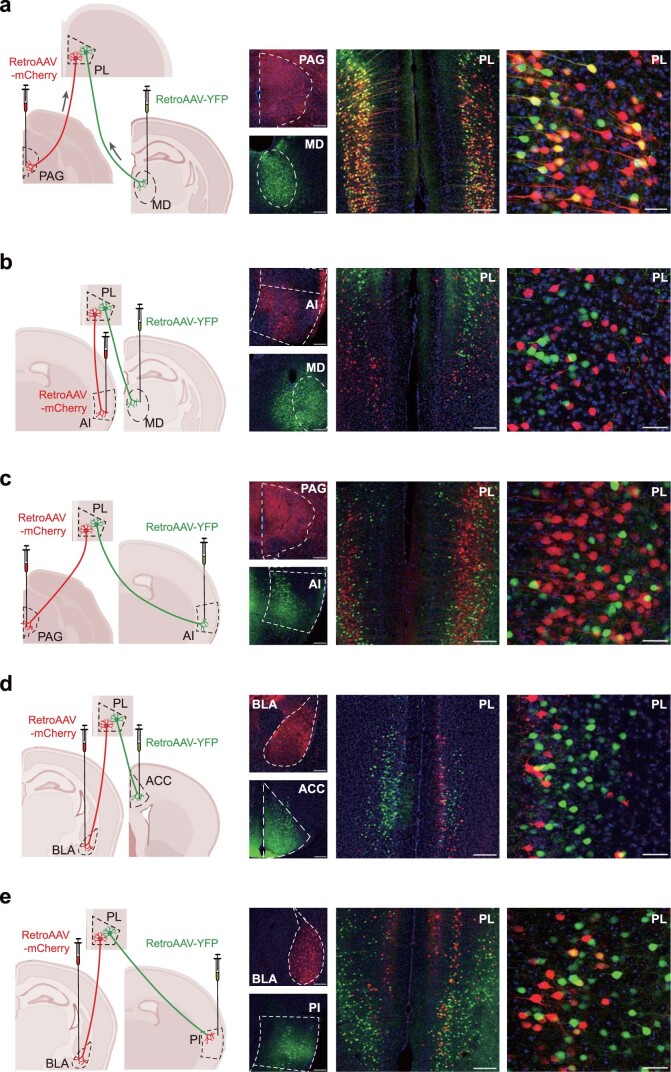
Comparison of spatial placement of prelimbic neurons projecting to diverse brain areas via retrograde labelling from projection areas. In panels a-e, the left panels show schematics of injections of YFP- or mCherry-expressing retro-AAV virions either in the indicated projection targets of prelimbic cortex (PL). Middle panels show the injection areas and the two panels on the right show example images from confocal microscopy revealing the juxtaposition or intermingling of PL neurons that project to these discrete and distant areas. The viral expression was bilaterally verified in n = 2 mice per group. Abbreviations: MD: mediodorsal thalamus; PAG: periaqueductal gray; AI: anterior insula; BLA: basolateral amygdala; ACC: rostral anterior cingulate cortex; PI: posterior insula. Scare bars represent 200 µm in the middle panels and 50 µm in panels on the right. Source data

We then studied the projections of tactile-responsive neurons and fear engram neurons in the brains of naive (sham) mice using quantitative analysis of fluorescent tags. A schematic of the experiments is shown in Fig. 4d and Extended Data Fig. 7a, a schematic for projection intensity analysis is shown in Extended Data Fig. 7b, data from individual mice are shown in Extended Data Fig. 6b, and average data are shown in Fig. 4e. Detailed statistical comparisons are shown in Supplementary Table 2. Compared to mice not exposed to fear conditioning, projections of tactile-responsive neurons in mice exposed to fear conditioning (Fig. 4e) showed increased connectivity to key components of the fear network^18,19^, such as the lateral amygdala (Extended Data Fig. 6c) and anterior cingulate cortex (Fig. 4f) and other centers important for emotion and negative affect in pain^1,8,9,15,16^, such as the agranular insular cortex (Extended Data Fig. 6c) and the mediodorsal thalamus (Fig. 4f)^15^, thus partly resembling the projection map of prefrontal fear memory engram neurons. A further increase in intensity of prefrontal tactile-responsive neuron projections to the mediodorsal thalamus was also seen in nerve-injured mice exposed to fear conditioning (Fig. 4e,f). Interestingly, the convergence of fear conditioning and neuropathic pain was associated with the strongest density of projections not only in the mediodorsal thalamus (Fig. 4f) but also in the paratenial nucleus (Extended Data Fig. 6c), which remains nearly entirely functionally unexplored and belongs to midline nuclei that have been suggested to play a role in retrieving consolidated fear conditioning via connectivity with amygdaloid nuclei^20^. These differences in projection patterns are not inconsistent with the observation of a lack of differences in representation of fear memory and pain across different prelimbic layers, because within a specific cortical layer, neighboring neurons can have very different projection patterns; we confirmed this in tracing analyses using retro-AAV injections in the diverse aforementioned projection areas of the prelimbic cortex and observed a juxtaposition or intermingling of prelimbic cortex neurons that project to these discrete and distant areas (Extended Data Fig. 8).

### Reversal of pathological nociceptive hypersensitivity by silencing of prefrontal long-term fear engrams

**Fig. 5:**
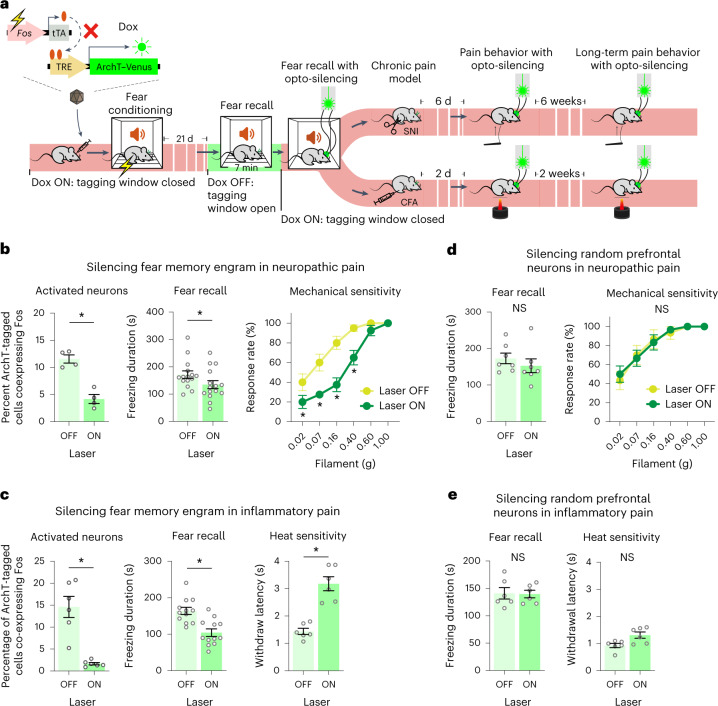
Disrupting long-term fear memory reduces established nociceptive hypersensitivity in inflammatory and neuropathic pain. **a**, Scheme of tagging prefrontal long-term fear memory engram neurons with ArchT and impact of optogenetic silencing of the fear memory engram on CFA-induced inflammatory pain and SNI-induced neuropathic pain. **b**,**c**, Activity-dependent expression of ArchT in prefrontal long-term fear engram neurons for light-induced silencing (left) of the specific neuronal population is shown via Fos immunohistochemistry; impact on fear recall (middle) and neuropathic mechanical hypersensitivity (at day 6 after SNI; **b**) or thermal hypersensitivity after paw inflammation (at day 2 after CFA; **c**) is shown. In **b** and **c**, an average of 4,844 neurons per mm^3^ and 4,084 neurons per mm^3^ were labeled with ArchT, respectively. **d**,**e**, Impact of optogenetically silencing a randomly targeted prelimbic neuronal population on fear recall and neuropathic mechanical hypersensitivity (**d**) or inflammatory thermal hypersensitivity (**e**). In **d** and **e**, an average of 3,147 neurons per mm^3^ and 2,575 neurons per mm^3^ were labeled with ArchT, respectively; *P < 0.05. Data for von Frey behavior were analyzed by two-way ANOVA with a Tukey’s multiple comparisons test. A paired *t*-test was used for freezing behavior and heat sensitivity, and an unpaired *t*-test was used for comparing overlapping populations. All data are shown as mean ± s.e.m. All *P* and *F* values are shown in Supplementary Table 1. Source data

**Extended Data Fig. 9:**
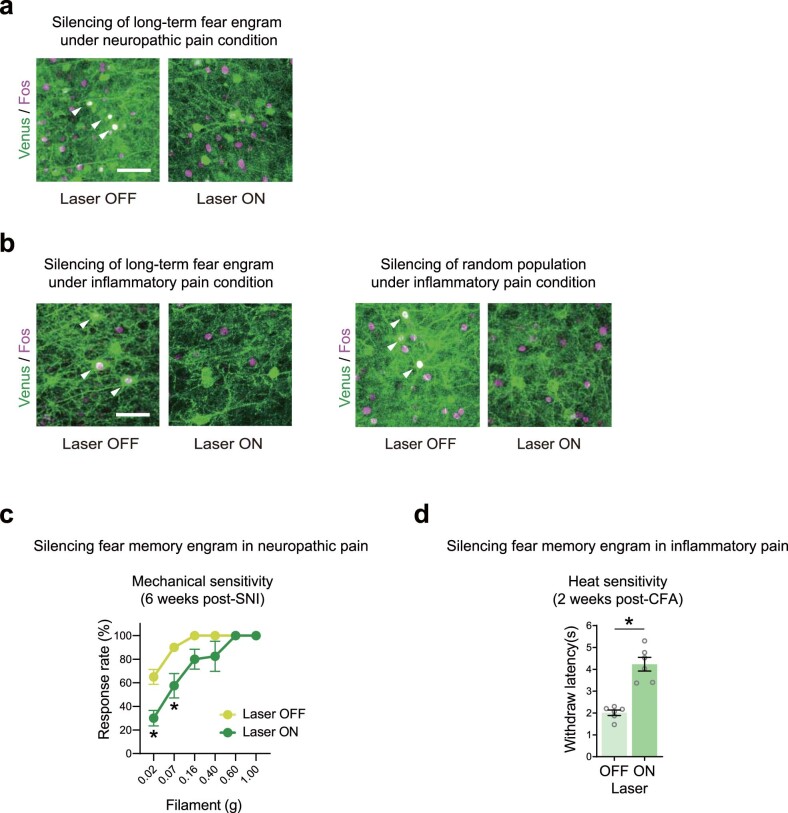
Controls for optogenetic manipulations and testing impact on pain-unrelated aversion in pathological pain. **(a, b)** Typical examples of Fos immunohistochemistry showing validation of optogenetic silencing of prefrontal fear memory engram in mice with nerve injury (a) or inflammatory pain (b, left panel) and silencing of random neurons by synapsin promoter-mediated expression of ArchT in a random population (b, right panel); the expression was verified and quantified in n = 4 mice/group; arrowheads indicate double-labelled neurons and scale bars represent 50 µm. (**c, d**) Impact of optogenetic silencing of prefrontal long-term fear memory engram on chronic stages of neuropathic mechanical hypersensitivity (6 weeks post-SNI, panel c) and inflammatory heat hyperalgesia (2 weeks post-CFA, panel d); two-way ANOVA with Tukey’s multiple comparisons test for von Frey behavior and paired *t*-test for heat sensitivity; * *p* < 0.05; n = 6 mice/group for CFA experiments. All data are shown as mean + /− S.E.M. All *p* and *F* values are shown in Supplementary Table 1. Source data

This enhanced recruitment of fear memory circuitry by tactile and nociceptive stimuli under chronic pain conditions implies a role for fear memory in hypersensitivity and predicts that perturbing established fear memory engrams will alleviate chronic pain. We directly tested these hypotheses by optogenetically silencing prefrontal engrams of long-term fear memory (Fig. 5a) and observed that tactile allodynia and hyperalgesia were reduced significantly when peak hypersensitivity was reached at 6 d after SNI (Fig. 5b and Extended Data Fig. 9a) and chronically established at 6 weeks after SNI (Extended Data Fig. 9c). Similarly, inflammatory thermal hypersensitivity was significantly and strongly reduced when mice were tested with yellow light illumination compared to without illumination at 2 d after CFA (Fig. 5c and Supplementary Video 3) and at 2 weeks after CFA (Extended Data Fig. 9d). By contrast, optogenetic silencing of a random population of prefrontal neurons did not lead to changes in neuropathic and inflammatory hypersensitivity (Fig. 5d,e and Extended Data Fig. 9b). These data thus show that chronically established neuropathic and inflammatory hypersensitivity can be significantly and specifically reversed after suppressing the recall of long-term fear memory.

## Discussion

**Extended Data Fig. 10:**
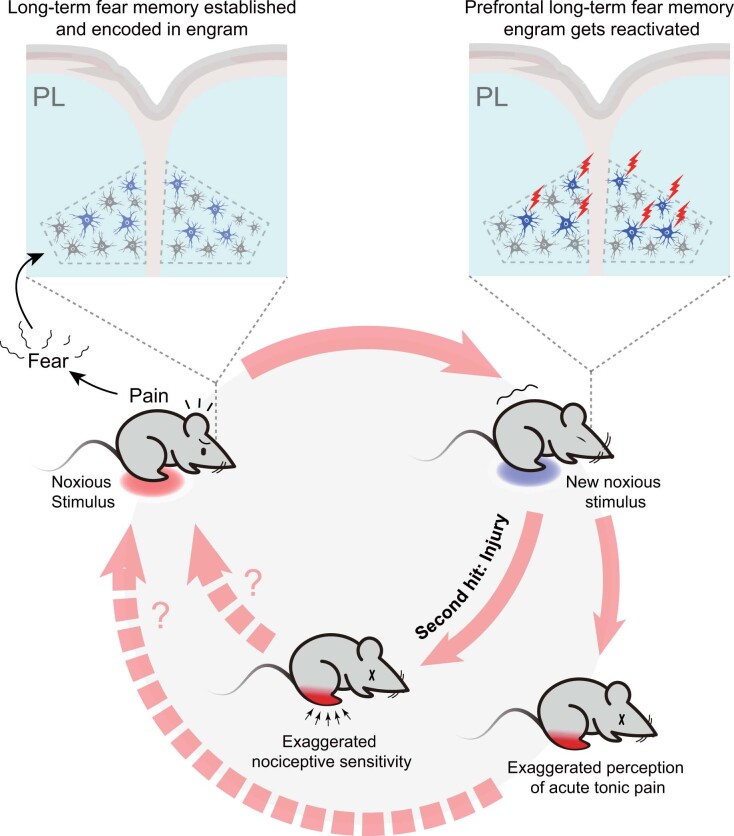
Prefrontal mechanistic basis of chronic pain-fear interaction. Model based on results of this study to explain the cellular basis of how long-term fear memory induced by a painful episode can alter the perception of tonic pain as well as nociceptive hypersensitivity in chronic neuropathic or inflammatory pain conditions at a remote point in life. Source data

This study reveals cellular mechanistic underpinnings for the concept emerging from macroscopic human imaging that emotion circuitry intersects with pain pathways to perpetuate pain^8,21–23^ (Extended Data Fig. 10). So far, synaptic potentiation and cellular sensitization have been suggested as mechanistic correlates of sensitization of pain perception triggered by prior exposure to noxious stimuli^17,24,25^. While the importance of these mechanisms is unequivocal, their short duration cannot explain why the experience of pain is altered over such large time frames in the clinical context. Moreover, the molecular players, such as excitatory receptors and signaling mediators, that have been suggested to underlie fear–pain interactions^24,25^ by virtue of being shared between diverse forms of memory and nociceptive sensitization^23^, are broadly expressed throughout the nervous system and mediate diverse functions. Here, we describe a unique mechanism that accounts for both long-term modulation and specificity, in that a painful experience encodes a fear memory that is stored in a discrete, specific cohort of prefrontal cortical neurons, which is subject to reactivation after exposure to a new pain-evoking stimulus at a later point in life and thereby intensifies pain perception. Surprisingly, in the presence of a ‘second hit’, such as a tissue injury, this modulation is extended toward nociception and even tactile hypersensitivity, thus revealing the cellular basis for how fear circuits can intersect with somatosensory circuits to foster hyperalgesia and the severely debilitating symptom of allodynia, respectively.

That activation of prelimbic fear memory engrams alone is both necessary and sufficient to simulate modulation of pain by long-term fear at an organismal behavioral level is interesting because several other areas of the fear network, such as the amygdala, the anterior cingulate cortex and the hippocampus, are also activated during pain states^25^. The importance of the prelimbic cortex might arise from its function in storage and recall of long-term associative memory. Previous studies that broadly targeted the prelimbic cortex have shown that activation, not silencing, of excitatory output suppresses neuropathic and inflammatory hypersensitivity^26,27^. By contrast, we showed that silencing of specific prelimbic fear memory neurons suppressed ongoing pain and pathological hypersensitivity. Our data suggest that the difference between these sets of functionally distinct neurons may lie in the denser connectivity of the prelimbic fear engram neurons with areas implicated in fear modulation, affect and mood compared to prelimbic neurons that receive somatosensory inputs. This supports the recently emerging notion that despite being intermingled in close physical proximity, individual cortical pyramidal neurons can have very different projection targets and engage in functionally distinct circuits^28^, for which we also show direct evidence here. A major limitation of this study, however, is that all manipulations of engrams were targeted to neurons that show an increase in activity in association with pain or fear. Silencing of ongoing activity of neurons is also an important component of neuronal encoding of functions and behaviors, but a lack of technological means of targeting such ongoing activity currently hampers the consideration of their contributions in fear–pain interactions in this study.

Our observations predict that fear memory, in turn, plays a role in manifestation of nociceptive and tactile hypersensitivity in neuropathic and inflammatory pain, and we experimentally validated this through optogenetic interventions. Our findings thus not only support the notion of using therapeutic strategies for extinguishing long-term fear memory in treating chronic pain (for example, via pharmacological and cognitive behavioral therapy approaches for fear extinction^29,30^) but go beyond to predict that targeting prefrontal mechanisms will lead to increased therapeutic value. This is conceivable with both site-specific delivery or uncaging of drugs and with closed-loop neurostimulation and neuromodulation interventions targeting the activity of prefrontal circuitry.

In summary, this study describes a mechanism that explains why long-term fear, instilled by prior exposure to pain, can perpetuate pain and predispose toward pain chronicity. Remarkably, this study reveals that a small, select population of prefrontal cortical circuitry is necessary and sufficient to mediate these interactions. Our study provides causal evidence for diminishing pathological pain by overpowering anticipatory fear and gives an impetus for developing interventions targeting prefrontal circuitry in individuals with chronic pain and comorbid fear.

## Methods

### Animals

All experimental procedures were approved by the local governing body (Regierungspräsidium Karlsruhe: Abteilung 3, Landwirtschaft, Ländlicher Raum, Veterinär und Lebensmittelwesen’, approval numbers 35-9185.81/G-205/18, 35-9185.81/G-119/14 and 35-9185.82/G-113/20), adhered to institutional guidelines and were performed abiding to German Law that regulates animal welfare and the protection of animals used for scientific purposes (TierSchG, TierSchVersV). C57BL/6J mice (25–30 g; 8–15 weeks old, male) were housed in groups of one to three mice per cage in a ventilation unit, with food and water provided ad libitum on a natural 12-h light/12-h dark cycle. Room temperature and humidity ranged from 20 to 23 °C and 40 to 60%. ARRIVE guidelines were followed. Our sample sizes are similar to those reported in previous publications. Based on previous studies, we determined the sample size using G-power analysis and therefore have a very clear set of what sample size is required for the behavioral and histochemical data reported. In all experiments, groups were randomized, and mice were allocated to experimental groups by a researcher different from the experimenter. Experimenters were blinded to the identity of the treatment groups. Animals were excluded that clearly looked unwell after surgery or in experiments involving viral expression and the injection and/or expression was not successful.

### Preparation of AAV vectors

The cloning method for the pAAV-cFos-tTA construct has been published^31^, and the plasmid is publicly available (Addgene, 66794). The *Fos* promoter is based on the construct used by Reijmers et al.^32^. For the creation of plasmid pAAV-6P-Cmini.iCRE-mCherry, pAAV-6P-SEWB^33^ was used as a backbone. The viral gene segment encoding the hSyn promoter, enhanced green fluorescent protein, the WPRE element and the BGH terminator signal of pAAV-6P-SEWB was replaced by the improved iCre recombinase^34^ and the red fluorescent protein mCherry under the tight control of the bidirectional tTA-dependent TRE promoter, as described previously^35^. The rAAV-hSyn-ArchT2A-Venus AAV1/AAV2 virions were a kind gift from R. Sprengel (Max Planck Institute for Medical Research Heidelberg University, Heidelberg, Germany)^36^. For generating the pAAV-TREtight-ArchT2A-Venus construct, the ~1.7-kilobase ArchT2A–Venus cassette was PCR amplified (forward primer with an AscI site: 5′-ATGCTATTTGGCGCGCCCGAGGCTGTGAGC-3′; reverse primer: 5′-CGGACCTAGTTCGAGTGCGGCCGCTTTACT-3′) and subcloned with AscI and BsrGI into parent vector pAAV-ITR-PTREtight-hM3Dq-mCHERRY-WPRE-pA-ITR (Addgene, 66795) to create the final pAAV-ITR-PTREtight-ArchT2A-Venus-WPRE-pA-ITR plasmid. The tTA-dependent ChR2 virus (AAV1/AAV2-ITR-PTREtight-ChR2-eYFP-WPRE-pA-ITR) was a kind gift from the laboratory of M. Fuhrmann (Deutsches Zentrum für Neurodegenerative Erkrankungen, Bonn, Germany)^37^.

Recombinant AAV1/AAV2 virions were generated by calcium phosphate cotransfection of HEK293 cells (Stratagene) with each of the above-mentioned plasmids and plasmids pDP1rs and pDP2rs (Plasmid Factory, 401 and 402). These constructs provided adenoviral helper functions and the AAV1 *rep*, AAV2 *rep* and AAV2 *cap* genes. Cells were lysed by freeze–thaw cycles and subjected to benzonase nuclease (Merck) digestion to retrieve the crude lysate. The rAAV1/rAAV2 particles were then purified via heparin-agarose (Merck) affinity chromatography and concentrated with Amicon filter tubes (Merck Millipore).

For the analysis of projection target regions, a combination of AAV1/AAV2-cFOS-tTA, AAV1/AAV2-iCre-mCherry and AAV5-Ef1a-DIO-hChR2(H134R)-EYFP (purchased from UNC Vector Core) at a ratio of 1:2:2 was injected. For retrograde tracing of projections starting in the prelimbic cortex, the two retrograde tracers hSyn1-eYFP (Addgene, 117382-AAVrg) and hSyn-mCherry (Addgene, 114472-AAVrg) were injected bilaterally in distinct target regions.

### Surgical procedures

Mice were generally anesthetized with intraperitoneal doses of fentanyl (Janssen-Cilag; 0.01 mg kg^−1^), medetomidine (Alvetra; 0.3 mg kg^−1^) and midazolam (Hameln Pharma Plus; 4 mg kg^−1^). Pedal reflex caused by a firm toe pinch was monitored regularly to ensure continued surgical plane of anesthesia.

With the origin set to bregma in a stereotaxic alignment system (David Kopf Instruments), viral injections were performed using a fine glass tip micropipette bilaterally into the prelimbic cortex (±0.25 mm lateral, +1.9 mm anterior and –1.35-mm depth from the pia or ±0.9 mm lateral, +1.94 mm anterior and –1.6-mm depth with a 15° sagittal tilt to the midline when implanting chronic optical fibers) with a flow rate of 25 nl min^−1^ and a total injection volume of 300–400 nl per side. For axonal projection analysis, only the right side was injected. For retrograde tracing, a volume of 20 nl per side and area was injected in the following brain areas: periaqueductal gray (±0.606 mm lateral, –4.48 mm anterior and –2.1-mm depth), mediodorsal thalamus (±0.48 mm lateral, –1.7 mm anterior and –3.21-mm depth), posterior insular cortex (±3.85 mm lateral, –0.34 mm anterior and –2.05-mm depth), anterior insular cortex (±3.1 mm lateral, +0.98 mm anterior and –2.6-mm depth), basolateral amygdala (±3.15 mm lateral, –1.31 mm anterior and –3.6-mm depth) and anterior cingulate cortex (±0.25 mm lateral, +1.18 mm anterior and –1.01-mm depth)^38^.

#### Optical fiber implantation

Optical fiber implants consisted of a ceramic ferrule (Thorlabs, CFLC230-10) fitted with an optical fiber (Ø = 200 µm; NA = 0.39 or 0.5; Thorlabs, FT200UMT or FP200URT, respectively) extending 1.9 mm from the ferrule base and were implanted immediately after the viral injections. The tip of the optical fiber was positioned 50 µm above the injection site, and the ceramic ferrule was fixed to the skull with Paladur dental cement (Heraeus).

#### Tetrode probe implantation

For electrophysiological experiments, a small craniotomy just lateral to the midline in the right hemisphere was performed, and the dura mater was removed. An eight-shank 64-channel silicon probe (NeuroNexus; Buzsaki64-5 mm-200-160 with H64LP-30 mm connector, mounted on a d-drive) was implanted at an initial depth of 0.75 mm from the pia, with the electrode contacts facing lateral in a parallel orientation to the midline, the longitudinal shank axis angled 17° in a caudal direction and the posterior-most shank positioned above the caudal pole of the prelimbic cortex (1.5 mm anterior to bregma, 0.45 mm lateral). A gold pin (0.5 mm Ø) implanted at a depth of 1 mm at the midline posterior to lambda was used as signal ground. The craniotomy area around the electrode shanks was filled with softened bone wax (World Precision Instruments), and the base of the d-drive was cemented with C&B super bond (Sun Medical) to the skull and embedded in several layers of Paladur dental cement, with the Omnetics connector positioned caudal to the electrode cover cap. Once fixed in place, electrodes were lowered to a depth of 1,250 µm.

#### SNI

Mice were anesthetized with 3% isoflurane, and the peroneal and tibial nerves were ligated and distally transected. Behavioral testing was performed between day 4 and week 6 after the operation^39^.

#### CFA model

Inflammatory pain-like behavior in mice was induced by intradermal injection of 20 μl of CFA into the hind paw (F5881, Sigma) under 3% isoflurane anesthesia.

### Behavioral tests

#### Capsaicin-induced tonic pain-related nocifensive behavior

A capsaicin (Sigma) injection solution was prepared at a concentration of 0.06% (wt/vol) in 10% DMSO (Thermo Fisher Scientific) and PBS (Thermo Fisher Scientific). Under 2% isoflurane anesthesia (Baxter), 20 µl of the capsaicin solution was injected into the hind paw. Mouse behavior was video recorded over a period of 5 min, and the total time the animal displayed nocifensive behavior (paw lifting, licking, flinching and writhing) was assessed.

#### Mechanical sensitivity, von Frey test

von Frey filaments with increasing forces (0.02 to 1 g, five applications per filament, each applied 30 s apart) were applied to the hindpaws. Mechanical thresholds were defined by the minimal filament force that elicited withdrawal behavior within 3 s of application in ≥60% of trials.

#### Heat sensitivity, Hargreaves test

Animals were left to acclimate for 15 min in the Hargreaves setup, the lasers were set to the ON or OFF condition, and withdrawal latencies in response to heat stimulation of the inflamed hind paw were measured using an infrared heat apparatus (Ugo Basile; 270 mW cm^−2^). Four measurements were taken for each animal using an interval of 5 min to avoid adaptation to the repeated heat stimulation.

#### Cued fear conditioning and fear memory recall testing

Before fear conditioning, all animals were habituated for 2 d to human experimenters. Two contexts with distinct visual cues were used. A neutral context A consisted of an unenclosed transparent chamber (20 cm × 20 cm) on a transparent acrylic glass floor. The shock-paired context B consisted of a transparent chamber (17 cm × 17 cm) with a parallel steel bar floor connected to a shock generator (Ugo Basile) in a 40 cm × 40 cm black box. Before each animal was placed in the chamber, context A was cleaned with 70% ethanol, and context B was cleaned with 2% benzyl alcohol in 70% ethanol. Baseline behavior was recorded over a period of 7 min following a 90-s acclimatization period for both contexts, and on the following day, each animal received two fear conditioning sessions in context B separated by 3–4 h. A fear conditioning session consisted of five tone-paired foot shocks presented at random intervals during a 7-min period. Each tone (5 kHz, 70 dB) lasted 30 s and was followed by a foot shock (0.5 mA, 1 s). For fear memory recall testing, the animals were placed again in context A (or context B) and were presented with the 5-kHz tone five times in 7 min without a foot shock, and the time spent freezing was scored. Baseline, conditioning and recall trials were recorded with ANY-maze software (Stoelting; v4.82 or v6.06) with the freezing detection threshold set to 400 ms.

#### Foot shock

Animals were introduced to the shock chamber in the absence of additional sensory cues, where they received five foot shocks (0.5 mA, 1 s) distributed over a 5-min period.

#### In vivo optical stimulation

For optical suppression in ArchT-expressing mice, we used a yellow diode laser with continuous illumination (*λ* = 589 nm, Shanghai Laser & Optics Century). The laser power output setting was measured with a calibrated power meter (Thorlabs, PM100D) and adjusted individually to obtain 1.5 mW at the tip of each optic fiber before the implant. Yellow laser illumination was constant over the length of the behavioral testing period. For optical stimulation of ChR2-expressing mice, we used a blue diode laser (*λ* = 473 nm, 8 mW) connected to a pulse generator for 20-Hz laser pulses with a pulse length of 10 ms. Yellow/blue light-filtered protective glasses (Thorlabs) were used by the experimenter for blinding during behavioral testing to avoid subjective judgement.

#### Reward learning

Performance in a reward task was tested using automated Bussey-Saksida Mouse Touch Screen operant chambers (Campden Instruments)^40^ and ABET II TOUCH software (Lafayette Instrument). Throughout training and testing stages, animals had limited access to water (30 min d^−1^). For habituation and training, procedures outlined in the ABET II TOUCH paired discrimination task module (version 3) were followed. Animals had to touch the monitor window when a cue was presented to obtain a 7-µl water reward. After an intertrial interval of 20 s, the animal could initiate a new trial with a nose-poke of the food tray. Incorrect window touches were punished with the house light being turned on with no reward presentation for 5 s, followed by the regular 20-s intertrial interval until the animal could initiate a new trial. A learning session lasted until the mouse initiated 30 trials or a maximum of 30 min. The animals were tested for the operant reward-seeking performance in the same task during two 30-min sessions with and without laser stimulation on consecutive days.

#### White noise place aversion test

In the real-time place aversion test to white noise^41^, the setup consisted of two chambers (15 cm × 15 cm each; chamber A: horizontally striped walls and cocoa scent; context B: vertically striped walls and berry scent) that were connected via a neutral middle chamber (8 cm × 8 cm) Following acclimatization and a 20-min baseline session, the chamber preferred (determined by the time spent in each chamber) during the baseline session was paired to white noise (90 dB) in two subsequent place aversion sessions with and without laser stimulation. Recording and tracking analysis were performed with the ANY-maze software (Stoelting).

#### Open field test

The open field test was performed on day 7 following fear engram labeling. The animal was attached to the fiberoptic patch cords, placed in the center of the 40 cm × 40 cm open field chamber and allowed to move freely within the open field chamber for a 10-min assessment period. The laser was turned OFF or ON either during the first or second 5-min period in a balanced fashion. Movement patterns were recorded via a video camera placed above the open field chamber and analyzed via ANY-maze software (Stoelting).

### Activity-dependent cell labeling

#### Labeling of two distinct states via expression of activity-based reporter and native Fos

For the activity-dependent dual labeling experiments and projection analyses, Dox administered via the drinking water (2 mg ml^−1^ in 5% sucrose) was removed for a period of 48 h, after which the animals were exposed to foot shock, capsaicin or the fear memory recall treatments to induce activity-driven mCherry expression. The mice were returned to the home cage, and Dox administration via the drinking water was resumed. After 24 h, the animals were presented with a second stimulus modality and were killed 90 min later to detect stimulus-induced native Fos expression along with mCherry reporter-labeled neurons in fixed brain sections. To induce endogenous Fos expression during neuropathic mechanical allodynia, the filament previously calculated to lead to ≥60% withdrawal (0.6 g, sham mice; 0.07 g, SNI mice) was applied every 30 s for 20 min. To induce endogenous Fos expression during inflammatory heat hyperalgesia, 10 Hargreaves stimuli were applied using a minimal interval of 1 min.

#### Optogenetic manipulation of the prelimbic remote fear memory engram

For experiments involving the activity-dependent ArchT or ChR2 constructs^42^, animals received 200 mg kg^−1^ Dox chow before surgery and underwent fear conditioning as mentioned above. Dox supplementation was tapered to 40 mg kg^−1^ for 48 h and was completely withdrawn for 3 d, after which neuronal engrams were labeled in a fear recall session on day 28 after fear conditioning, and Dox supplementation with 200 mg kg^−1^ chow was resumed. At days 2 and 3 after ArchT/ChR2 labeling, animals underwent two more fear memory recall tests with and without laser stimulation. On days 4 and 5 after ArchT labeling, capsaicin behavior was tested with and without laser stimulation^42^. In experiments testing the influence of the remote fear memory engram on chronic pain-like behavior, SNI surgeries or CFA paw injections were performed 2 d after remote fear labeling. Cued fear behavior and mechanical sensitivity were then tested each on alternate days with and without optogenetically manipulating the remote fear engram. The sequence of laser ON and laser OFF treatment sessions were balanced across both testing days. On the final test day, half of the animals were randomly assigned to a laser ON group and half were assigned to a laser OFF group (corresponding to behavioral assessment in the absence or presence of optogenetic modulation), exposed to a stimulus (that is, capsaicin, CFA injection or mechanical stimulation) and perfused with formalin fixative 90 min following testing; brains were processed for Fos immunofluorescence.

#### TMT exposure

For testing the effect of innate fear on pain perception, TMT^43,44^ was pipetted on a 2 cm × 2 cm filter paper and placed in an exposure chamber. Baseline freezing without TMT exposure was recorded for 10 min. Following Dox withdrawal, mice were placed in a transparent testing box (20 cm × 20 cm) for 10 min while exposed to TMT odor. During the trial (but not recall), five tones (5 kHz, 70 dB, 30 s) were played at random intervals. To test fear behavior after activation of the labeled engram, freezing was recorded for 10 min on days 3 and 4 after labeling, with and without optogenetic activation. Baseline, TMT exposure and optogenetic trials were recorded with a firewire camera (UniBrain) and ANY-maze software (Stoelting) with the freezing detection threshold set to 400 ms. On days 5 and 6 after labeling, capsaicin behavior was tested with and without laser stimulation. On the final test day, animals were perfused with formalin fixative 90 min following testing, and brains were processed for Fos immunofluorescence.

### In vivo electrophysiology

Neural signals were amplified and digitized with an RHD 2164 head stage, transmitted via a motor-assisted hybrid rotary joint (Doric Lenses) to a RHD2000 USB interface board and acquired on a PC using the RHD2000 interface software (Intan Technologies). Signals were digitized at 30 kHz for a bandwidth from 0.1 to 7,500 Hz. Behavioral recording sessions were performed in two blocks 28 to 32 d following the initial day of fear conditioning. Animals first underwent a tone-cued fear retrieval session (five times for 30 s over a 7-min period), and after a home cage period (3–4 h), brain activity was recorded again for 5 min in an unstimulated neutral context. Immediately after, animals received a subcutaneous capsaicin injection (20 µl, 0.06% dissolved with 10% DMSO in PBS) in the plantar surface of the hind paw and were placed back in the neutral context chamber for a further 5-min recording period. Nocifensive behavior was captured with a video camera and ANY-maze software. Animals received five more tone–shock pairings the following day to prevent fear extinction, electrodes were lowered by 250 µm, and tone-cued fear, unstimulated neutral context and acute pain behavior was assessed again 2 d later. Capsaicin was always injected in the alternate and never the same hind paw. In control experiments, animals were reexposed to the neutral baseline context or underwent a second fear retrieval session with the electrodes left in place 3–4 h later. Tone periods and freezing events were recorded via digital input channels of the RHD2000 USB interface board. Nociceptive behavior was assessed offline by an operator recording onset and offset times for nocifensive episodes (paw lifting, licking, flinching and writhing). The timing of nocifensive video and brain activity data was aligned by matching up key sporadically occurring freezing episodes. Electrode positions were identified post hoc in formalin-fixed brain sections immunolabeled for glial fibrillary acidic protein and cell nuclei stained with Hoechst reagent. Only channel data from electrode shanks positioned in the dorsomedial prefrontal cortex were used for the analysis.

### Electrophysiology data analysis

Data analysis was performed in MATLAB R2014a and R2017a (MathWorks). Intan data files were converted to .mat files using a modified version of the Opensource import function supplied by Intan. Session files of the same experimental block or the same state control (baseline–baseline and repeated tone-cued fear sessions) were concatenated into a single file. The median of all 64 channels was subtracted from each channel to remove systemic noise and artifacts. The signal was then bandpass filtered from 300 to 6,000 Hz (fourth order Butterworth), and spike times and waveforms for spikes with an absolute amplitude greater than 9 × median absolute deviation were extracted. Single-unit spike times were then isolated by configuring the upper and lower four channels of each shaft as a group and using a WaterShed spike sorting function written by Alexei Koulakov (Cold Spring Harbor Laboratory). Isolated units typically contained <0.5% interspike intervals in the refractory period (<2 ms) and had spike amplitudes that followed a normal distribution and were stable over time and across sessions. To avoid dual detection of units that registered on neighboring channels of the upper and lower cluster groups of a shaft, pairs of units with common spike times of >10% were identified, and the unit with the higher proportion of spikes in the refractory period was removed.

To estimate the variance in unit firing rate changes observed on a repeated encounter of the same state, we isolated units from eight distinct experiments with either the unstimulated neutral context (*n* = 3, 105 units) or the tone-cued fear retrieval session (*n* = 5, 110 units). Mean firing rates for each unit were calculated for both the repeated baseline (4 min) and fear retrieval (7 min) sessions. The ratio of the mean firing rates from the second to the first session was log transformed, and all units were pooled to obtain a normal distribution centered on 0. The standard deviation of this same-state data set was used to set a threshold of 2 × s.d. to identify units with a significant change in global firing rate for cued fear and/or acute pain compared to the unstimulated baseline state within an experimental block for all three sessions. Mean global firing rates were calculated over a period of 4 min for the unstimulated baseline and 7 min for the cued fear sessions and for the initial 3 min after the capsaicin injection for the acute pain session. To detect units with firing rate changes matched to behavioral episodes, the mean spike rate was calculated with a sliding window (0.8 s wide, 0.4-s interval), and the binned firing rates of the cued fear and acute pain sessions were assigned into two groups based on window bin onset and offset times falling within or outside a freezing or nocifensive behavioral episode, respectively. Differences in the mean firing rate between two groups were then assessed with a randomized permutation test^45^ comparing the behavior-matched firing rates of each unit to the binned firing rates of the unstimulated baseline period. Units that had significantly increased behavior-matched firing rates (*P* < 0.001) with an effect size of >0.5 were then assigned into pain- or fear-specific, common pain- and fear-coding or pain- and fear-unspecific types. Binned firing rates of the cued fear and acute pain sessions were normalized to the mean firing rate of the unstimulated baseline session for visualization in heat map plots. To detect putative interneurons and principal neurons in the data set, we performed an unsupervised linkage analysis of two waveform parameters (half-amplitude duration and trough to peak time) and mean firing rate using Euclidean distances and Ward’s method on the *z*-scored variables for all units to obtain the two most prominent clusters^46^.

### Tissue processing and immunofluorescence

Animals were perfused transcardially with 50 ml of PBS, followed by 50 ml of 10% formalin (Merck). Brains were dissected and postfixed in 10% formalin overnight at 4 °C, and 50-µm coronal sections were collected with a vibratome (Leica, VT100S). Sections were processed in 20 mM sodium citrate (AppliChem) solution at 85 °C for 20 min, rinsed with 50 mM glycine (AppliChem) for 10 min and treated with 5% normal donkey serum (Abcam) in PBST (PBS + 0.2% Triton X-100) for 60 min and incubated with primary antibody at 4 °C overnight. Sections were then rinsed twice with 10% horse serum in PBS for 10 min, incubated with secondary antibodies (1:700) for 1 h and washed twice with PBS before incubating with Hoechst 33342 (1:10,000, Molecular Probes) for 10 min. Sections were washed for 10 min in 10 mM Tris-HCl (Carl Roth) before mounting with Mowiol (Carl Roth). For SOM and VIP co-staining, sections were incubated with primary antibodies at 4 °C for 6 d, washed three times with 5% horse serum in PBST for 15 min and incubated with secondary antibodies at 4 °C overnight.

Primary antibodies were used at the following dilutions: rabbit anti-Fos (1:1,000, Synaptic Systems, 226003), guinea pig anti-SATB2 (1:500, Synaptic Systems, 327004), mouse anti-TLE4 (1:500, Santa Cruz, sc365406), rabbit anti-LHX2 (1:500, Millipore Sigma, ABE1402), rat anti-CTIP2 (1:200, Abcam, ab18465), guinea pig anti-parvalbumin (1:1,000, Swant, GP72), rat anti-SOM (1:300, EMD Millipore, MA5-16987) and rabbit anti-VIP (1:700, Abcam, ab8556). Secondary antibodies were goat anti-rabbit Alexa 405 (Invitrogen, A-31556), goat anti-guinea pig Alexa 488/647 (Invitrogen, A-11073/A-21206), donkey anti-rabbit Alexa 488/594 (Invitrogen, A-21206/A-21207), donkey anti-mouse Alexa 488/647 (Invitrogen, A-21202/A-31571) and donkey anti-rat Alexa 488 (Invitrogen, A-21208), respectively.

### Image acquisition and quantification

Sections were imaged with an LCS SP8 confocal microscope (Leica). Images were acquired under a ×20 objective (Leica, HC PL APO) with identical parameters. Stacked images were maximally projected and localized with the corresponding atlas section^38^ to determine the region of interest. Animals with virus overexpressed were excluded. ImageJ (version 2.1.0, National Institutes of Health) was used, and experimenters were blinded during analyzing.

### Automated DAPI counting and chance calculation

Automated nuclei counting was performed using ilastik pixel classification probability map combined with the Cellpose pretrained model ‘CP’^47^. The pixel classification workflow was trained using two pixel labels on six to ten raw DAPI images in advance. The generated probability maps were segmented in three dimensions (3D), and chance level of overlap for Fos and mCherry labeling was calculated as (total number of Fos^+^/total number of DAPI) × (total number of mCherry^+^/total number of DAPI) × 100% (refs. ^32,48^). The same was applied for ChR2^+^ or ArchT^+^ neurons.

### Imaging and analysis of axonal projections

Sections including target regions (anterior to bregma: 1.7 to 2.4 mm for prelimbic cortex, 1.93 to 0.73 mm for nucleus accumbens, 1.93 to −1.23 mm for insular, −0.35 to −1.91 mm for thalamus, −0.71 to −1.79 mm for amygdala and −2.91 to −4.71 for periaqueductal gray, respectively) were selected based on the Allen Brain atlas (available from http://atlas.brain-map.org/). Widefield images were acquired on DAPI and YFP channels to screen sections under an epifluorescent microscope (Nikon). YFP intensity-coded colormaps were generated to identify brain regions with YFP fluorescence using an ImageJ/Fiji script (CreateIntensityColorMaps_V0.ijm)^49^. The intensity of each pixel in the images was calculated and valued as the following: 0 was set for a value between 0 and 10, 10 was set for a value between 10 and 20 and so on, while 50 was set for a value between 50 and 100, 100 was set for a value between 100 and 150, 150 was set for a value between 150 and 200, and 200 was set for a value between 200 and 255. The ImageJ/Fiji ‘glasbey inverted’ lookup table was applied to visualize intensity change. Colormaps and raw widefield images were registered with the BUnwarpJ plugin (https://imagej.net/plugins/bunwarpj/) and Paxinos Brain Atlas^38^ in an ImageJ/Fiji script (https://github.com/cberri/2D_Registration_BrainAtlas_ImageJ-Fiji)^49^ to describe the brain regions with high YFP intensities.

Regions with high-intensity colormap values were imaged with a confocal microscope at a resolution of 1,024 × 1,024 and a depth of 20 µm under the ×20 objective. The nuclear YFP signal was set as YFP background. The DAPI channel was segmented in 3D using the StarDist deep learning tool^50^, which was trained using around 300 labeled nuclei in a customized Jupyter Notebook. Ground truth nuclear labels in stacks were annotated in ImageJ/Fiji with the LabKit plugin^51^. An ImageJ/Fiji script (BackgroundSubtractionStarDist_Mask.ijm) uses the 3D ImageJ Suite plugin^52^ to estimate the intensity value of the YFP signal in each segmented nucleus in 3D and calculate the average values within all the nuclei in the stack as the background. A maximum intensity projection (MIP) was computed for each of the 3D YFP background-subtracted stacks and saved.

Average intensities of each MIP were extracted by a customized ImageJ/Fiji script (RegionSelection.ijm), which allows for the detection of different intensity features (mean, standard deviation, mode, median, skew, kurt, minimum, maximum, area and intden; ImageJ/Fiji, Set Measurements plugin). Fluorescence intensity was used to evaluate the quality of the injection and expression. Mice with a mean intensity not significantly higher than the home cage mice were excluded, as were the mice with a mean intensity above 2 s.d. of the average intensity in the experimental group. Mean MIP intensities were measured and analyzed. MATLAB (2019a) was used to generate heat maps for the mean intensity data.

### Statistical analysis

Prism (version 8.0) was used for statistical analysis of all cell count and behavioral test data. MATLAB was used for statistical analysis of all electrophysiological data as described in the Electrophysiology data analysis section above. One-way and two-way analysis of variance (ANOVA), Mann–Whitney *U*-test, Student’s *t*-test and a randomization test^45^ were used as indicated, and multiple comparison testing was used where appropriate. The data met the assumptions of the statistical tests used, and normality and equal variances were formally tested. All data are expressed as mean ± s.e.m. unless stated otherwise. In all tests, a value of *P* < 0.05 was considered significant.

### Reporting summary

Further information on research design is available in the Nature Portfolio Reporting Summary linked to this article.

## Online content

Any methods, additional references, Nature Portfolio reporting summaries, source data, extended data, supplementary information, acknowledgements, peer review information; details of author contributions and competing interests; and statements of data and code availability are available at 10.1038/s41593-023-01291-x.

## Supplementary information

Supplementary Information Supplementary Table 1

Reporting Summary

Supplementary Video 1 Nocifensive behavior indicative of tonic, ongoing pain after capsaicin injection unilaterally in the left hind paw is pronounced when the laser is OFF, that is, when there is no silencing of the long-term fear memory engram.

Supplementary Video 2 Nocifensive behavior indicative of tonic, ongoing pain after capsaicin injection unilaterally in the left hind paw is less pronounced when the laser is ON, that is, after bilateral yellow laser light-induced and ArchT-mediated neuronal silencing of the long-term fear memory engram in the mouse prefrontal cortex.

Supplementary Video 3 Hypersensitivity to heat in mice with paw inflammation, measured via latency to respond to heat under laser OFF conditions.

Supplementary Video 4 Reduction of hypersensitivity to heat in mice with paw inflammation, seen as longer latency to respond to heat, after ArchT-mediated silencing of the prefrontal long-term fear memory engram compared to laser OFF conditions.

Supplementary Table 2 Excel sheet with detailed information on numerous statistical comparisons in data corresponding to projection analyses.

## Source data

Source Data Statistical Source Data for Figs. 1–5 and Extended Data Figs. 1–10.

## Data Availability

The spike time data of the electrophysiological experiments are available via the heiDATA repository at 10.11588/data/VEEIDP. Databases/datasets used in the study include Allen Institute for Brain Science (2004), Allen Mouse Brain Atlas [Coronal Atlas], available from mouse.brain-map.org, and Allen Institute for Brain Science (2011). Source data are provided with this paper.

## Code availability

MATLAB scripts used to analyze the electrophysiological data are available together with the spike time data in the same heiDATA repository at 10.11588/data/VEEIDP.
