# Author Correction: Prefrontal engrams of long-term fear memory perpetuate pain perception

**DOI:** 10.1038/s41593-026-02386-x

**Published:** 2026-07-06

**Authors:** Alina Stegemann, Sheng Liu, Oscar Andrés Retana Romero, Manfred Josef Oswald, Yechao Han, Carlo Antonio Beretta, Zheng Gan, Linette Liqi Tan, William Wisden, Johannes Gräff, Rohini Kuner

**Affiliations:** 1 Institute of Pharmacology, Heidelberg University, Heidelberg, Germany; 2 Department of Life Sciences, Faculty of Natural Sciences, Imperial College London, London, UK; 3 Laboratory of Neuroepigenetics, Brain Mind Institute, School of Life Sciences, École Polytechnique Fédérale de Lausanne (EPFL), Lausanne, Switzerland

**Keywords:** Neural circuits, Fear conditioning

Correction to: *Nature Neuroscience* 10.1038/s41593-023-01291-x, published online 6 April 2023.

In the version of the article initially published, the “Inflammatory pain (CFA)” image in Extended Data Fig. 5e was duplicated from the “Laser ON” image in Extended Data Fig. 4d. Extended Data Fig. 5e has now been amended with an image from the correct experimental group in the HTML and PDF versions of the article.

