# Supplementary material for: Prefrontal engrams of long-term fear memory perpetuate pain perception
Source: Nat Neurosci. 2023 Apr 6;26(5):820–9. doi: 10.1038/s41593-023-01291-x (PMC10166861; doi:10.1038/s41593-023-01291-x)
Supplement: Supplementary file 1 — Supplementary Table 1 [file 41593_2023_1291_MOESM1_ESM.pdf]

nature neuroscience

Article

https://doi.org/10.1038/s41593-023-01291-x

Prefrontal engrams of long-term fear memory perpetuate pain perception

In the format provided by the authors and unedited

Supplementary table 1: Detailed information on statistical parameters for all comparisons in main figures and Extended Data figures.

Figure	Group name	F and P values	Stats test
Fig. 1b	Common cells	F (3, 15) = 28.32, P < 0.0001<br>Foot Shock (stimulus 1) followed by Foot Shock (stimulus 2) vs Capsaicin (stimulus 1) followed by Fear Recall (stimulus 2) P < 0.0001<br>Foot Shock (stimulus 1) followed by Foot Shock (stimulus 2) vs Fear recall (stimulus 1) followed by Capsaicin (stimulus 2) P < 0.0001<br>Fear Recall (stimulus 1) followed by Fear Recall (stimulus 2) vs Capsaicin (stimulus 1) followed by Fear Recall (stimulus 2) P = 0.0004<br>Fear Recall (stimulus 1) followed by Fear Recall (stimulus 2) vs Fear recall (stimulus 1) followed by Capsaicin (stimulus 2) P = 0.0404	One-way ANOVA with Tukey's multiple comparisons
Fig. 1c	Proportion of common glutamatergic cells	F (3, 6) = 17.09, P = 0.0024<br>SATB2 p=0.0034<br>LHX2 p=0.9636<br>Ctip2 p=0.0458<br>TLE4 p=0.9087	Two-way ANOVA with Šidák correction for multiple comparisons
	Proportion of common gabaergic cells	F (2, 4) = 11.10, P = 0.0233<br>SOM p=0.0263<br>PV p=0.8112<br>VIP p=0.0224	
Fig. 1e	Activated neurons	P = 0.0270	Unpaired t-test, two-tailed
	Fear recall	P = 0.0045	Paired t-test, two-tailed
	Tonic pain	P = 0.0002	
Fig. 1f	Activated neurons	P < 0.0001	Unpaired t-test
	Fear recall	P = 0.0069	Paired t-test
	Tonic pain	P = 0.0019	
Fig. 2b	Proportion of all units with behavior locked firing increase	Fear/Pain Modality: F(1, 6)=5.799, P=0.0527<br>Incr-/Decreased Activity: F(2, 12)=33.50, P<0.0001<br>Interaction Factor: F(2, 12)=8.09, P =0.0060	Two-way ANOVA with Sidak's multiple comparisons test
Fig. 2c	Proportion of putative principal neurons with behavior locked firing increase	Fear/Pain Modality: F(1, 6)=6.531, P=0.0432<br>Incr-/Decreased Activity: F(2, 12)=28.09, P<0.0001<br>Interaction Factor: F(2, 12)= 7.00, P=0.0097	

	Proportion of putative interneurons with behavior locked firing increase	Fear/Pain Modality: $F(1, 5) = 1.305$, $P=0.305$<br>Incr-/Decreased Activity: $F(2,10)=0.1023$, $P=0.904$<br>Interaction Factor: $F(2,10)=0.7078$, $P=0.516$	
Fig. 2d	Proportion of units with behavior locked firing increase	P = 0.6778	Unpaired t-test
Fig. 3a	Silencing of random prefrontal neurons: Fear recall	P = 0.077	Paired t-test, two-tailed
	Silencing of random prefrontal neurons: Tonic pain	P = 0.839	
	Activation of random prefrontal neurons: Fear recall	P = 0.2591	
	Activation of random prefrontal neurons: Tonic pain	P = 0.3792	
Fig. 3b	White noise Fear recall engram	$F(2, 27) = 18.00$, $P < 0.0001$<br>240 s: Laser off vs Baseline P = 0.2357<br>240 s: Laser on vs Baseline P = 0.1922<br>480 s: Laser off vs Baseline P = 0.0364<br>480 s: Laser on vs Baseline P = 0.0002<br>720 s: Laser off vs Baseline P = 0.0004<br>720 s: Laser on vs Baseline P < 0.0001<br>960 s: Laser off vs Baseline P = 0.004<br>960 s: Laser on vs Baseline P < 0.0001<br>1200 s: Laser off vs Baseline P < 0.0001<br>1200 s: Laser on vs Baseline P = 0.0001	Two-way ANOVA with Šidák correction for multiple comparisons
	White noise Random neurons	$F(2, 30) = 15.84$, $P < 0.0001$<br>240 s: Laser off vs Baseline P = 0.0557<br>240 s: Laser on vs Baseline P = 0.2828<br>480 s: Laser off vs Baseline P = 0.0003<br>480 s: Laser on vs Baseline P = 0.0009<br>720 s: Laser off vs Baseline P = 0.0089<br>720 s: Laser on vs Baseline P = 0.0081<br>960 s: Laser off vs Baseline P = 0.0029<br>960 s: Laser on vs Baseline P = 0.0010	

		1200 s: Laser off vs Baseline P < 0.0001<br>1200 s: Laser on vs Baseline P = 0.0026	
Fig. 3c	Reward learning	F (5, 72) = 4.956,<br>P = 0.0006	One-way ANOVA
	Reward test: Number of responding trials	P = 0.4583	Paired t-test, two-tailed
	Reward test: % accuracy	P = 0.204	Wilcoxon test
Fig. 3d	Innate fear: Freezing duration	P = 0.048	Paired t-test, two-tailed
	Tonic pain: Duration of pain behavior	P = 0.0197	
Fig. 4a	Inflammatory heat hyperalgesia	F (1, 12) = 15.74,<br>P = 0.0006<br>Post fear conditioning p=0.4283<br>2 Days post CFA p=0.0006	Two-way ANOVA with Sidak's test for multiple comparisons
	Neuropathic mechanical allodynia	F (3, 168) = 130.6,<br>P < 0,0001<br>0.02g filament p=0.9921<br>0.07g filament p>0.9999<br>0.16g filament p=0.2019<br>0.40g filament p=0.0120<br>0.60g filament p=0.0273<br>1.00g filament p=0.4948	Two-way ANOVA with Tukey's multiple comparisons
Fig. 4b	Overlap between thermal nociception and fear memory	P < 0.0001	Unpaired t-test, one-tailed
Fig. 4c	Overlap between tactile sensitivity and fear memory	P = 0.0022	
Fig. 4f	Projections	F (148, 370) = 1.733,<br>P<0.0001<br>ACC:<br>Sham no FC vs Sham FC<br>p=0.0449<br>MD:<br>Fear vs Sham FC p=0.039<br>Fear vs SNI FC p=<0.0001<br>Sham no FC Sham FC p=0.0265<br>Sham FC vs SNI FC p=0.0185<br>SNI no FC vs SNI FC p=0.0066<br>*For remaining p values: please see Supplementary data 1*	Two-way ANOVA with Tukey's test for multiple comparisons
Fig. 5b	Activated neurons	P = 0.0006	Unpaired t-test
	Fear recall	P = 0.0044	Paired t-test
	Mechanical sensitivity	F (5, 35) = 7.245, P<0.0001<br>0.02g filament p=0.0117<br>0.07g filament p<0.0001<br>0.16g filament p<0.0001<br>0.40g filament p<0.0001<br>0.60g filament p=0.7706<br>1.00g filament p>0.9999	Two-way ANOVA with Tukey's multiple comparisons test
Fig. 5c	Activated neurons	P < 0.0001	Unpaired t-test
	Fear recall	P = 0.0009	Paired t-test
	Heat sensitivity	P = 0.0015	
Fig. 5d	Fear recall	P = 0.0644	
	Mechanical sensitivity	F (5, 25) = 0.7093,<br>P = 0.6220	Two-way ANOVA with Tukey's

		0.02g filament p=0.6538<br>0.07g filament p=0.9798<br>0.16g filament p=0.9798<br>0.40g filament p=0.9798<br>0.60g filament p>0.9999<br>1.00g filament p>0.9999	multiple comparisons test
Fig. 5e	Fear recall	P = 0.9177	Paired t-test
	Heat sensitivity	P = 0.0638	
ED Fig. 1b	Prelimbic neurons tagged in homecage	P <0.0001	Unpaired t-test, two-tailed
ED Fig. 1d	Tagging of prelimbic neurons	F (3, 19) = 4.087,<br>P = 0.0213	One-way ANOVA with Dunnet's multiple comparisions
ED Fig. 2a	Time course of Doxycycline withdrawal	F (2, 28) = 16.86,<br>P < 0.0001<br>72 h: Homecage vs Capsaicin P = 0.0019	Two-way ANOVA with Sidak's test for multiple comparisons
ED Fig. 2c	Distance	P = 0.578465	Paired t-test, two-tailed
	Speed	P = 0.569085	
ED Fig. 2d	Distance	P = 0.6007	
	Speed	P = 0.2118	
ED Fig. 5a	Baseline mechanical sensitivity	F (3, 360) = 0.6322,<br>P = 0.5947<br>0.02g filament:<br>Pre conditioning uninjured paw vs pre conditioning injured paw p>0.9999<br>Pre conditioning uninjured paw vs post conditioning uninjured paw p>0.9999<br>Pre conditioning uninjured paw vs post conditioning injured paw p>0.9999<br>Pre conditioning injured paw vs post conditioning uninjured paw p>0.9999<br>Pre conditioning injured paw vs post conditioning injured paw p>0.9999<br>Post conditioning uninjured paw vs post conditioning injured paw p>0.9999<br>0.07g filament:<br>Pre conditioning uninjured paw vs pre conditioning injured paw p=0.931<br>Pre conditioning uninjured paw vs post conditioning uninjured paw p>0.9999<br>Pre conditioning uninjured paw vs post conditioning injured paw p=0.7464<br>Pre conditioning injured paw vs post conditioning uninjured paw p=0.931	Two-way ANOVA with Tukey's multiple comparisons test

		Pre conditioning injured paw vs post conditioning injured paw p=0.978<br>Post conditioning uninjured paw vs post conditioning injured paw p=0.7464<br>0.16g filament:<br>Pre conditioning uninjured paw vs pre conditioning injured paw p=0.978<br>Pre conditioning uninjured paw vs post conditioning uninjured paw p=0.4955<br>Pre conditioning uninjured paw vs post conditioning injured paw p=0.3751<br>Pre conditioning injured paw vs post conditioning uninjured paw p=0.7464<br>Pre conditioning injured paw vs post conditioning injured paw p=0.6232<br>Post conditioning uninjured paw vs post conditioning injured paw p=0.9971<br>0.4g filament:<br>Pre conditioning uninjured paw vs pre conditioning injured paw p=0.931<br>Pre conditioning uninjured paw vs post conditioning uninjured paw p=0.4955<br>Pre conditioning uninjured paw vs post conditioning injured paw p=0.931<br>Pre conditioning injured paw vs post conditioning uninjured paw p=0.8524<br>Pre conditioning injured paw vs post conditioning injured paw p>0,9999<br>Post conditioning uninjured paw vs post conditioning injured paw p=0.8524<br>0.6g filament:<br>Pre conditioning uninjured paw vs pre conditioning injured paw p=0.9971<br>Pre conditioning uninjured paw vs post conditioning uninjured paw p=0.8524<br>Pre conditioning uninjured paw vs post conditioning injured paw p=0.931<br>Pre conditioning injured paw vs post conditioning uninjured paw p=0.7464	

		Pre conditioning injured paw vs post conditioning injured paw p=0.8524<br>Post conditioning uninjured paw vs post conditioning injured paw p=0.9971<br>1.0 g filament:<br>Pre conditioning uninjured paw vs pre conditioning injured paw p=0.8524<br>Pre conditioning uninjured paw vs post conditioning uninjured paw p>0.9999<br>Pre conditioning uninjured paw vs post conditioning injured paw p=0.931<br>Pre conditioning injured paw vs post conditioning uninjured paw p=0.8524<br>Pre conditioning injured paw vs post conditioning injured paw p=0.9971<br>Post conditioning uninjured paw vs post conditioning injured paw p=0.931	
ED Fig. 5b	Neuropathic mechanical allodynia	F (1, 84) = 3.132,<br>P = 0.0804<br>0.02g filament p=0.9998<br>0.07g filament p=0.7729<br>0.16g filament p=0.7729<br>0.40g filament p=0.7729<br>0.60g filament p=0.9998<br>1.00g filament p>0.9999	
ED Fig. 5c	Inflammatory heat hyperalgesia	F (1, 10) = 1.352,<br>P = 0.2719<br>Post Fear Conditioning p=0.8760<br>2 Days post CFA p=0.9764	
**ED Fig. 6a**	Neurochemical identity of prelimbic neurons in SNI and Sham condition	F (4, 8) = 160.5,<br>P<0.0001<br>SATB2 p=0.9881<br>Ctip2 p=0.9935<br>TLE4 p=0.9256<br>SOM p>0.9999<br>PV p=0.7347	
ED Fig. 6c	Projections	F (148, 370) = 1.733,<br>P<0.0001<br>AIP:<br>Sham no FC vs Sham FC p=0,004<br>LA:<br>Sham no FC vs Sham FC p= 0,0379<br>PT:<br>Fear recall vs. SNI FC p <0,0001<br>Sham FC vs. SNI FC p= 0,0021<br>SNI no FC vs. SNI FC p <0,0001<br>*For remaining p values: please see Supplementary data 1*	Two-way ANOVA with Tukey's test for multiple comparisons

ED Fig. 9c	Mechanical sensitivity (6 weeks post-SNI)	F (5, 35) = 4.391,<br>P = 0.0033<br>0.02g filament p=0,0002<br>0.07g filament p=0,0004<br>0.16g filament p=0,0531<br>0.40g filament p=0,1193<br>0.60g filament p>0,9999<br>1.00g filament p>0.9999	Two-way ANOVA with Tukey's multiple comparisons test
ED Fig. 9d	Heat sensitivity (2 weeks post-CFA)	P = 0.0016	Paired t-test
