# Supplementary material for: Prefrontal engrams of long-term fear memory perpetuate pain perception
Source: Nat Neurosci. 2023 Apr 6;26(5):820–9. doi: 10.1038/s41593-023-01291-x (PMC10166861; doi:10.1038/s41593-023-01291-x)
Supplement: Supplementary file 2 — Reporting Summary [file 41593_2023_1291_MOESM2_ESM.pdf]

nature portfolio

Corresponding author(s): Prof. Dr. Rohini Kuner

Last updated by author(s): 29 Jan, 2023

Reporting Summary

Nature Portfolio wishes to improve the reproducibility of the work that we publish. This form provides structure for consistency and transparency in reporting. For further information on Nature Portfolio policies, see our Editorial Policies and the Editorial Policy Checklist.

Statistics

For all statistical analyses, confirm that the following items are present in the figure legend, table legend, main text, or Methods section.

n/a	Confirmed	
☐	☒	The exact sample size (*n*) for each experimental group/condition, given as a discrete number and unit of measurement
☐	☒	A statement on whether measurements were taken from distinct samples or whether the same sample was measured repeatedly
☐	☒	The statistical test(s) used AND whether they are one- or two-sided<br>*Only common tests should be described solely by name; describe more complex techniques in the Methods section.*
☒	☐	A description of all covariates tested
☐	☒	A description of any assumptions or corrections, such as tests of normality and adjustment for multiple comparisons
☐	☒	A full description of the statistical parameters including central tendency (e.g. means) or other basic estimates (e.g. regression coefficient) AND variation (e.g. standard deviation) or associated estimates of uncertainty (e.g. confidence intervals)
☐	☒	For null hypothesis testing, the test statistic (e.g. *F*, *t*, *r*) with confidence intervals, effect sizes, degrees of freedom and *P* value noted<br>*Give P values as exact values whenever suitable.*
☒	☐	For Bayesian analysis, information on the choice of priors and Markov chain Monte Carlo settings
☒	☐	For hierarchical and complex designs, identification of the appropriate level for tests and full reporting of outcomes
☒	☐	Estimates of effect sizes (e.g. Cohen's *d*, Pearson's *r*), indicating how they were calculated

Our web collection on statistics for biologists contains articles on many of the points above.

Software and code

Policy information about availability of computer code

Data collection	Any-Maze video tracking software (Stoelting Co, v4.82 and v6.06); Leica Application Suite X (LAS X, v3.3.0); RHD2000 interface software (Intan Technologies, LLC, v1.3); ABET II TOUCH software (Lafayette Instrument, IN, USA v2.15)
Data analysis	ImageJ software (National Institutes of Health, USA v1.50b and v2.1.0,); Matlab R2014a and R2017a (MathWorks). WaterShed spike sorting function written by Alexei Koulakov (Cold Spring Harbor Laboratory, Cold Spring Harbor, NY). GraphPad Prism (GraphPad Software, San Diego, California USA v8.0.0)<br><br>Image J/Fiji and Matlab analysis scripts associated with the manuscript are deposited along with the electrophysiology dataset in a repository (heiDATA, 2023) with accession code https://doi.org/10.11588/data/VEEIDP .

For manuscripts utilizing custom algorithms or software that are central to the research but not yet described in published literature, software must be made available to editors and reviewers. We strongly encourage code deposition in a community repository (e.g. GitHub). See the Nature Portfolio guidelines for submitting code & software for further information.

Data

Policy information about availability of data

All manuscripts must include a data availability statement. This statement should provide the following information, where applicable:

- Accession codes, unique identifiers, or web links for publicly available datasets
- A description of any restrictions on data availability
- For clinical datasets or third party data, please ensure that the statement adheres to our policy

Databases/datasets used in the study: Allen Institute for Brain Science (2004). Allen Mouse Brain Atlas [dataset]. Available from mouse.brain-map.org. Allen Institute for Brain Science (2011).

Data availability: Individual data points shown in figures are provided in a source data file with this manuscript. The spike-time data of the electrophysiological experiments are available via the heiDATA repository at the following URL: https://doi.org/10.11588/data/VEEIDP

Code availability: Matlab scripts used to analyze the electrophysiological data are available together with the spike time data in the same heiDATA repository with a public accession code: https://doi.org/10.11588/data/VEEIDP

Human research participants

Policy information about studies involving human research participants and Sex and Gender in Research.

Reporting on sex and gender	N/A
Population characteristics	N/A
Recruitment	N/A
Ethics oversight	N/A

Note that full information on the approval of the study protocol must also be provided in the manuscript.

Field-specific reporting

Please select the one below that is the best fit for your research. If you are not sure, read the appropriate sections before making your selection.

☒ Life sciences ☐ Behavioural & social sciences ☐ Ecological, evolutionary & environmental sciences

For a reference copy of the document with all sections, see nature.com/documents/nr-reporting-summary-flat.pdf

Life sciences study design

All studies must disclose on these points even when the disclosure is negative.

Sample size

Our sample sizes are similar to those reported in previous publications. Based on previous studies we determined the sample size using G-power analysis and therefore have a very clear set of what sample size is required for the behavioral and histochemical data reported.

Exemplary references:
Gan, Z., Gangadharan, V., Liu, S., Korber, C., Tan, L. L., Li, H., Oswald, M. J., Kang, J., Martin-Cortecero, J., Mannich, D., Groh, A., Kuner, T., Wieland, S. and Kuner, R. (2022). Layer-specific pain relief pathways originating from primary motor cortex. Science 378 (6626), 1336-1343, doi: 10.1126/science.add4391.

Gangadharan, V., Zheng, H., Taberner, F. J., Landry, J., Nees, T. A., Pistolic, J., Agarwal, N., Mannich, D., Benes, V., Helmstaedter, M., Ommer, B., Lechner, S. G., Kuner, T. and Kuner, R. (2022). Neuropathic pain caused by miswiring and abnormal end organ targeting. Nature 606 (7912), 137-145, doi: 10.1038/s41586-022-04777-z.

Hu, X., Agarwal, N., Zhang, M. D., Ernfors, P., Kuner, R., Nyengaard, J. R. and Karlsson, P. (2022). Identification and quantification of nociceptive Schwann cells in mice with and without Streptozotocin-induced diabetes. J Chem Neuroanat 123, 102118, doi: 10.1016/j.jchemneu.2022.102118.

Li, H., Gan, Z., Wang, L., Oswald, M. J. and Kuner, R. (2022). Prolonged Suppression of Neuropathic Hypersensitivity upon Neurostimulation of the Posterior Insula in Mice. Cells 11 (20), doi: 10.3390/cells11203303.

Oswald, M. J., Han, Y., Li, H., Marashli, S., Oglo, D. N., Ojha, B., Naser, P. V., Gan, Z. and Kuner, R. (2022). Cholinergic basal forebrain nucleus of Meynert regulates chronic pain-like behavior via modulation of the prelimbic cortex. Nat Commun 13 (1), 5014, doi: 10.1038/s41467-022-32558-9.

Data exclusions	For analysis of experiments with brain injections, mice were excluded if the injection did not reach the target area or leaked beyond the target area, or if the expression was undetectable. Exclusion criteria were pre-established.
Replication	All experiments were successfully replicated at least once with several animals. The precise animal numbers are given in the figure legends.
Randomization	Groups were randomized and mice were allocated to experimental groups by a researcher different from the experimenter.
Blinding	Experimenters were always blinded to the identity of the treatment groups.

Reporting for specific materials, systems and methods

We require information from authors about some types of materials, experimental systems and methods used in many studies. Here, indicate whether each material, system or method listed is relevant to your study. If you are not sure if a list item applies to your research, read the appropriate section before selecting a response.

Materials & experimental systems

n/a	Involved in the study	
☐	☒	Antibodies
☐	☒	Eukaryotic cell lines
☒	☐	Palaeontology and archaeology
☐	☒	Animals and other organisms
☒	☐	Clinical data
☒	☐	Dual use research of concern

Methods

n/a	Involved in the study	
☒	☐	ChIP-seq
☒	☐	Flow cytometry
☒	☐	MRI-based neuroimaging

Antibodies

Antibodies used

Antibodies used were against Fos (Synaptic Systems, 226003; 1:1,000 dilution, host: rabbit), SATB2 (Synaptic Systems, 327004; 1:500 dilution, host: guinea pig), TLE4 (Santa Cruz, sc365406; 1:500 dilution, host: mouse), LHX2 (Millipore Sigma, ABE1402; dilution 1:500, host: rabbit), Ctip2 (Abeam, ab18465; 1:200 dilution, host: rat), parvalbumin (Swant, GP72; 1:1,000 dilution, host: guinea pig), SOM (EMD Millipore, MA5-16987; 1:300 dilution, host: rat), VIP (Abeam, ab8556; 1:700 dilution, host: rabbit).
Secondary antibodies used were Goat anti-rabbit Alexa 405 (lnvitrogen, A-31556) 1:700 dilution, Donkey anti-rabbit Alexa 488 (lnvitrogen, A-21206) 1:700 dilution, Donkey anti-mouse Alexa 488 (lnvitrogen, A-21202) 1:700 dilution, Donkey anti-rat Alexa 488 (lnvitrogen, A-21208) 1:700 dilution, Goat anti-guinea pig Alexa 488 (lnvitrogen, A-11073) 1:700 dilution, Donkey anti-rabbit Alexa 594 (lnvitrogen, A-21207) 1:700 dilution, Goat anti-guinea pig Alexa 647 (lnvitrogen, A-21206) 1:700 dilution and Donkey anti-mouse Alexa 647 (lnvitrogen, A-31571) 1:700 dilution. Specificity of the antibody staining was tested by omitting the primary antibody.

Validation

All the primary antibodies were used in non-living tissue for immunohistochemistry (IHC). These antibodies are extensively used for IHC purpose by the scientific community with numerous species-relevant citations for each primary antibody available on the manufacturers website. We routinely performed negative controls by omitting primary antibodies.

Primary antibodies manufacturer's validation statement:
- Fos (Synaptic Systems, 226003): "Reacts with: human (P01100), rat (P12841), mouse (P01101), monkey, ape, cow, dog, pig. Other species not tested yet. Specific for c-Fos." (https://sysy.com/product-factsheet/SySy_226003)

- SATB2 (Synaptic Systems, 327004): "Reacts with: rat, mouse (Q8VI24). Other species not tested yet. Applications: WB: not recommended; IP: not tested yet; ICC: 1 : 500; IHC: 1 : 200; IHC_P: 1 : 500" (https://sysy.com/product-factsheet/SySy_327004)

- TLE4 (Santa Cruz, sc365406): "TLE4 (E-10) is recommended for detection of TLE4 of mouse, rat and human origin by Western Blotting (starting dilution 1:100, dilution range 1:100 - 1:1000), immunoprecipitation [...], immunofluorescence (starting dilution 1:50, dilution range 1:50-1:500), immunohistochemistry (including paraffin-embedded sections) (starting dilution 1:50, dilution range 1:50-1:500) and solid phase ELISA (starting dilution 1:30, dilution range 1:30-1:3000). Suitable for use as control antibody for TLE4 siRNA (h): sc-38562, TLE3 siRNA (m): sc-36684, TLE4 shRNA Plasmid (h): sc-38562-SH, TLE3 shRNA Plasmid (m): sc-36684-SH, TLE4 shRNA (h) Lentiviral Particles: sc-38562-V and TLE3 shRNA (m) Lentiviral Particles: sc-36684-V. TLE4 (E-10) X TransCruz antibody is recommended for Gel Supershift and ChIP applications. Molecular Weight (predicted) of TLE4 isoforms 1/2/3: 84/77/88 kDa. Molecular Weight (observed) of TLE4: 95 kDa. Positive Controls: F9 cell lysate: sc-2245, SH-SY5Y cell lysate: sc-3812 or P19 cell lysate: sc-24760." (https://datasheets.scbt.com/sc-365406.pdf)

- LHX2 (Millipore Sigma, ABE1402): "Immunofluorescence Analysis: A representative lot detected Lhx2 immunoreactivity by fluorescent immunohistochemistry using OCT-embedded, PFA-fixed backskin cryosections from wild-type, but not Lhx2 knockout, mice at varies developmental stages, including E16.5, P0, P30, and the second telogen (Folgueras, A.R., et. al. (2013). Cell Stem Cell. 13(3):314-327)." (https://www.merckmillipore.com/DE/de/product/Anti-LHX2-Antibody,MM_NF-ABE1402)

- Ctip2 (Abcam, ab18465): "Specificity Detects 2 bands representing Ctip2 at about 120kD. Ctip2 is highly expressed in brain and in malignant T-cell lines derived from patients with adult T-cell leukemia/lymphoma. Tested applications Suitable for: ICC/IF, WB, Flow Cyt. Species reactivity Reacts with: Mouse, Human" (https://www.abcam.com/ctip2-antibody-25b6-ab18465.pdf)

- Parvalbumin (Swant, GP72): "This antibody was raised against recombinant mouse parvalbumin. GP72 reacts specifically with

parvalbumin on immunoblots of extracts of tissue originating from human, monkey, guinea pig, rabbit, rat, mouse and chicken (Fig. 1). The antibody specifically localizes parvalbumin using free-floating or mounted sections of brain (Fig. 2), kidney and muscles of probably all vertebrates The antiserum does not stain the brain of parvalbumin-KO mice. Spectacular staining found in immunohistochemistry on floating sections of 4% PFAfixed specimens. Working dilutions: Immunohistochemistry: 1:3'000 - 1:10'000, when performed with the avidin-biotin method." (https://www.swant.com/pdfs/GP72_Guinea_pig_anti_parvalbumin.pdf)

- SOM (EMD Millipore, MA5-16987): "Species Reactivity Guinea pig, Human, Mouse, Pig, Rat." (https://www.thermofisher.com/antibody/product/Somatostatin-Antibody-clone-YC7-Monoclonal/MA5-16987). Reference for Immunohistochemistry (PFA-fixed): Chhabra, N. F., Amend, A. L., Bastidas-Ponce, A., Sabrautzki, S., Tarquis-Medina, M., Sachs, S., Rubey, M., Lorenz-Depiereux, B., Feuchtinger, A., Bakhti, M., Lickert, H., Przemeck, G. K. H. and Hrabe de Angelis, M. (2021). A point mutation in the Pdia6 gene results in loss of pancreatic beta-cell identity causing overt diabetes. Mol Metab 54, 101334, doi: 10.1016/j.molmet.2021.101334.

- VIP (Abcam, ab8556): "Specificity This antibody reacts with a 3 kD vasointestinal peptide (VIP) localized in nerve fibers in the central and peripheral nervous system. The VIP producing tumors are usually neuroblastomas of endocrine tumors in the pancreas. Tested applications Suitable for: IHC-P, IP, IHC-FoFr, WB, ICC. Species reactivity Reacts with: Mouse, Rat, Human. General notes This antibody detects ganglion cells in both the superficial and deep plexus of the wall of the small bowel in human." (https://www.abcam.com/vip-antibody-ab8556.html)

Eukaryotic cell lines

Policy information about cell lines and Sex and Gender in Research

Cell line source(s)

HEK293 Cell line (AAV-293 cells, Stratagene, 240073)

Authentication

The HEK293 cell line was obtained via a commercial source. The manufacturer states that the provided cell content was determined by morphology, trypan-blue dye exclusion, and viable cell count.

Mycoplasma contamination

The HEK 293 cell line underwent mycoplasma detection by PCR and was negative.

Commonly misidentified lines
(See ICLAC register)

N/A

Animals and other research organisms

Policy information about studies involving animals; ARRIVE guidelines recommended for reporting animal research, and Sex and Gender in Research

Laboratory animals

Adult (8-34 weeks) C57BL/6J male mice (25 - 30 g) of wild-type were used in this study. Mice were housed in groups of 1-3 per cage (in ventilation unit) with food and water ad libitum on a 12 h light / 12 h dark cycle. Room temparature and humidity were ranging from 20-23 •c and 40-60%.

Wild animals

The study did not involve wild animals.

Reporting on sex

This study involved only male mice.

Field-collected samples

No field collected samples were used this study.

Ethics oversight

All of the animal experiments were conducted according to the ethical guidelines of 'Protection of Animals Act' under supervision of the 'Animal Welfare Officers' of Heidelberg University and were approved by the local governing body named 'Regierungsprasidium Karlsruhe: Abteilung 3 - Landwirtschaft, Landlicher Raum, Veterinar- und Lebensmittelwesen', Germany (Approval numbers: G-205/18, G-119/14 and G-113/20). ARRIVE guidelines were followed.

Note that full information on the approval of the study protocol must also be provided in the manuscript.
